# Allylation of Functionalized Aldehydes by Potassium Allyltrifluoroborate Catalyzed by 18-*Crown*-6 in Aqueous Media

**DOI:** 10.3390/molecules171214099

**Published:** 2012-11-28

**Authors:** Fernanda C. G. Barbosa, Juliano C. R. Freitas, Caio F. Melo, Paulo H. Menezes, Roberta A. Oliveira

**Affiliations:** Departamento de Química Fundamental, Universidade Federal de Pernambuco, Av. Jornalista Aníbal Fernandes, s/n - Cidade Universitária, Recife – PE, 50740-560, Brazil

**Keywords:** potassium allyltrifluoroborate, allylation, 18-*crown*-6

## Abstract

An efficient method for the allylation of aldehydes containing a broad range of functional groups using potassium allyltrifluoroborate is described. The reaction utilizes a catalytic amount of 18-*C*-6 in biphasic media under open atmosphere and room temperature to provide the corresponding homoallylic alcohols in high yields and without the necessity of any subsequent purification.

## 1. Introduction

The addition of allylic organometallics to aldehydes is an important method for C-C bond formation [1], while the product obtained from this reaction, an homoallylic alcohol, is an analogue of the compound obtained from the aldol addition of metal enolates [2]. In addition, the introduced alkene moiety can be readily converted into the corresponding aldehyde for further carbon-carbon homologation; or can be selectively epoxidized to introduce a new chiral center [3]. 

The general approaches to prepare homoallylic alcohols are based on the use of an allylic organometallic (e.g., Li [4], Mg [5], Mn [6], Zn [7], Pb [8], Bi [9], Ce [10], In [11], Sn [12] and many others [13,14,15,16,17]), organometalloid derivatives (e.g., B [18,19,20], Si [21]) and electrochemical based methods [22]. However, in some cases, these reagents are expensive and difficult to handle due to their Lewis base character. Alternative methods based on the Barbier reaction were also developed [23,24]. These methods avoid the previous preparation of the allylic organometallics, however, the use of zero valent metals can cause metal oxide (or hydroxide) precipitation and Rieke metals and some chlorosilanes are also sensitive to the reaction media. 

Organoboron compounds have proved to be very useful in the allylation of carbonyl compounds because of their easy formation and stability and to date, several methods for the allylation of carbonyl compounds employing allyl boronic acids or boronic esters were developed [18,19,20].

Potassium allyltrifluoroborates have proven to be a good option to replace allylboronic acids and allylboronate esters in allylation reactions, providing many advantages over the latter reagents [25,26,27,28]. These compounds are stable, easy to handle, compatible with several functional groups and can be stored for a long time. Several methods for the allylation of carbonyl compounds using allylic trifluoroborates promoted by a variety of Lewis acids [29,30,31] or palladium-catalysts [32] have been described. However, the search for an easy and reliable method for the allylation of aldehydes, that could be performed in an open flask with substrates containing a broad range of functional groups would be desirable, while some of the previously described methods for the allylation of aldehydes present drawbacks such as the price of catalysts [33] and the need to use anhydrous conditions [34]. 

In addition, the development of methods focusing on environmentally benign reaction media has become particularly prominent [35,36] and the use of water as (co)-solvent, which would seem to be the best option due to its simplicity and very low cost, is still a challenge. 

Crown ethers have the ability to promote reactions by phase-transfer catalysis solubilizing inorganic salts in apolar solvents. Because of their remarkable binding properties, many reactions can be promoted by using these compounds as catalysts. In addition, the study of crown ethers has greatly contributed to the development of host-guest chemistry and supramolecular chemistry [37]. 

Herein, we wish to describe an environmentally benign reaction for the synthesis of homoallylic alcohols based on the reaction of potassium allyltrifluoroborate and aldehydes containing different functional groups in aqueous media catalyzed by 18-*crown*-6.

## 2. Results and Discussion

Because our initial studies were focused on the development of an optimum set of reaction conditions, the reaction of 4-nitrobenzaldehyde (**1a**) and potassium allyltrifluoroborate (**2**) was examined to optimize these conditions. Therefore, **1a** (1 mmol) and **2** (1.2 mmol) were treated at room temperature with the appropriate catalyst (10 mol%) using different CH_2_Cl_2_-H_2_O ratios, and the progress of the reaction was monitored by TLC. The results are presented in Table 1 (all reported yields in this and other tables are isolated yields).

When a 1:1 mixture of CH_2_Cl_2_–H_2_O was used as the reaction solvent, product **3a** was obtained in good yields, even when the amount of the catalyst was decreased (Table 1, entries 2 and 3). However, with the variation in the amount of 18-*C*-6, longer reaction times were required to promote the reaction (Table 2, entries 1 and 2). Further decreasing in the amount of catalyst required even longer reaction times and gave a lower yield.

**Table 1:** Effect of solvent and catalyst on the allylation of 4-nitrobenzaldehyde (**1a**) by potassium allyltrifluoroborate **2**.

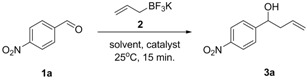			
	Catalyst (mol%)	Solvent	3a (%)
1	18-*C*-6 (1)	CH_2_Cl_2_:H_2_O	20
2	18-*C*-6 (5)	CH_2_Cl_2_:H_2_O	90^a^
3	18-*C*-6 (10)	CH_2_Cl_2_:H_2_O	94
4	18-*C*-6 (10)	CH_2_Cl_2_	15
5	18-*C*-6 (10)	H_2_O	80 ^a^
6	15-*C*-5 (10)	CH_2_Cl_2_:H_2_O	12
7	-	CH_2_Cl_2_:H_2_O	7

^a^ Yield obtained after 30 min of reaction.

**Table 2:** Allylation of aldehydes **1** with potassium allyl trifluoroborate **2** catalyzed by 18-*C*-6 (10 mol%).

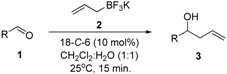					
	RCHO		Product		Yield (%)
1	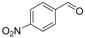	**1a**	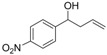	**3a**	94
2	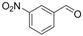	**1b**	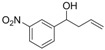	**3b**	82
3	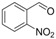	**1c**	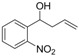	**3c**	80
4	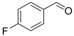	**1d**	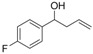	**3d**	87
5	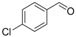	**1e**	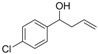	**3e**	85
6	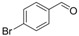	**1f**	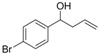	**3f**	86
7	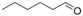	**1g**	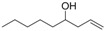	**3g**	89
8	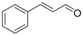	**1h**	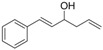	**3h**	90
9	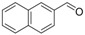	**1i**	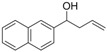	**3i**	87
10	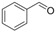	**1j**	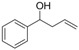	**3j**	88
11	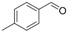	**1k**	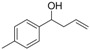	**3k**	82
12	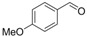	**1l**	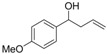	**3l**	89
13	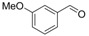	**1m**	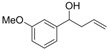	**3m**	90
14	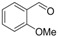	**1n**	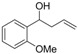	**3n**	91
15	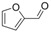	**1o**	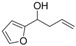	**3o**	86

When only CH_2_Cl_2_ was used as the reaction solvent, lower yields were obtained in the allylation reaction, probably due to the low solubility of **2** in this solvent (Table 1, entry 4). A similar behavior was observed when only water was used as the reaction solvent, again due to the low solubility of the aldehyde in water (Table 1, entry 5). In the absence of 18-*C*-6, the product was obtained only in 7% yield after 15 min (Table 1, entry 7) and when 15-*C*-5 was used as catalyst, lower yields were observed (Table 1, entry 6). This fact may be explained by the role of cation solvation by the ligand in size match-selectivity phenomena, which is widely accepted as the most important factor in controlling metal ion selectivity for macrocyclic ligands.

With the optimized reaction conditions, we next explored the generality of our method, by extending the conditions to different aldehydes. The results are listed in Table 2. In all cases, the reaction took place smoothly to give the corresponding homoallylic alcohols in good yields.

Various differently substituted aldehydes were used in the reaction. The allylation of aldehydes containing other electron-withdrawing groups gave the corresponding products in high yields (Table 2, entries 1–6). For example, when 2-, 3- and 4-nitrobenzaldehyde were used, similar yields were observed, indicating that the substituent position has a little influence in the reaction (Table 2, entries 1–3). Aldehydes containing electron-withdrawing groups such as 4-fluorobenzaldehyde (Table 2, entry 4); 4-bromobenzaldehyde (Table 2, entry 5) and 4-chlorobenzaldehyde (Table 2, entry 6) also gave similar yields. Other aromatic aldehydes such as β-naphtaldehyde (Table 2, entry 9), benzaldehyde (Table 2, entry 10) and 4-methylbenzaldehyde (Table 2, entry 11) also gave the homoallylic alcohols in high yields. When an α,β-unsaturated aldehyde was used, the corresponding 1,2-addition product was obtained exclusively, proving that the reaction is regioselective (Table 2, entry 8). For aliphatic aldehydes, the 18-*C*-6-catalyzed allylation also exhibited high efficiency (Table 2, entry 7). Electron-rich aldehydes reacted in good yields without influence of the substituent location (Table 2, entries 12–14). When a heterocyclic aldehyde reacted under the optimized conditions, the corresponding homoallylic alcohol was obtained in good yield (Table 2, entry 15). We attribute the small differences in the isolated yields to the different solubilities of reactants and products.

As we can see, this new method has several advantages in comparison to other previously described approaches. First of all, some methods are based on the use of allyl organometallics. It is known that these compounds are sometimes not suitable for carbonyl additions due to the deprotonation of the acidic alpha protons. Because of that the use of nonenolizable aldehydes is required. In addition, to avoid the Wurtz coupling, the preparation of allylic Grignard reagents requires low temperature and concentration [38].

The use of less reactive compounds such as allylsilanes [21] and allylstannanes [12] requires Lewis acids as additives. Although the utility of allylstannanes is further indicated by the commercial availability of some of them, the toxicity of these compounds makes them inappropriate for the use in pharmaceutical synthesis [39]. In addition, the removal of tributyltin residues from reaction mixtures is also a major issue. 

On the other hand, among the various allylboron reagents, the use of potassium allyltrifluoroborate seems to be the best option due to its stability, which also allows the complete characterization of these salts by heteronuclear NMR analysis [40], and exact mass measurements [41]. Additionally, organotrifluoroborate salts appear to have low toxicity [42].

Previously described methods for the allylation of aldehydes based on the conversion of potassium allyltrifluoroborate into the corresponding air and moisture sensitive allyl difluoroborane using BF_3_∙Et_2_O [34], or palladium catalysts [32] were reported. However, the use of potassium allyltrifluoroborate as the allylating agent without any previous transformation is an important advantage since it can be used in aqueous media and open atmosphere.

**Scheme 1:**
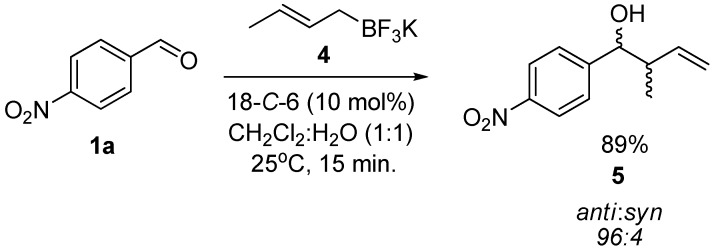
Crotylation of 4-nitrobenzaldehyde.

Furthermore, the use of potassium (*E*)-crotyltrifluoroborate (**4**) gave selectively the corresponding *anti* product **5** in a 96:4 *anti*:*syn* ratio, proving that the reaction was also diastereoselective (Scheme 1).

**Scheme 2:**
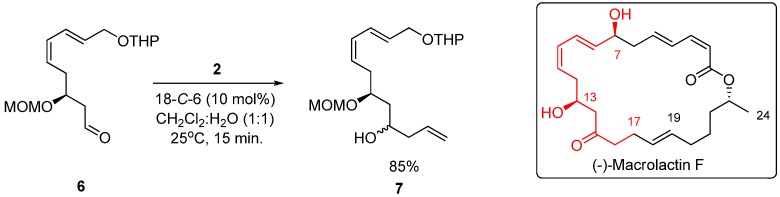
Synthesis of *C7*-*C17* fragment of (−)-macrolactin F.

Finally, to exemplify the utility of the proposed method in the synthesis of natural products, we applied it to the synthesis of *C7*-*C17* fragment of (−)-macrolactin F [43,44], a 24-membered macrolactone isolated from a marine *Bacillus* sp. Sc026 which exhibited antibacterial activity against *Bacillus subtilis* and *Staphylococcus aureus* (Scheme 2).

## 3. Experimental

### 3.1. General

^1^H-NMR and ^13^C-NMR data were recorded in CDCl_3_ on a Varian Unity Plus 300 spectrometer. The chemical shifts are reported as delta (δ) units in parts per million (ppm) relative to the solvent residual peak as the internal reference. Reactions were monitored by thin-layer chromatography on 0.25 mm E. Merck silica gel 60 plates (F254) using UV light, vanillin and *p*-anisaldehyde as visualizing agents. Potassium allyltrifluoroborate [45] and potassium (*E*)-crotyltrifluoroborate [34] were prepared according to the literature procedures.

### 3.2. General Procedure for the Allylation of Aldehydes ***1a**–**o*** with Potassium Allyltrifluoroborate ***(2)*** Catalyzed by 18-C-6

To a solution of the appropriate aldehyde **1a**–**o** (1.0 mmol) in CH_2_Cl_2_ (2 mL) was added 18-*C*-6 (26 mg, 10 mol%) followed by potassium allyltrifluoroborate (**2**, 177 mg, 1.20 mmol) and water (2 mL). The biphasic mixture was stirred for 15 minutes, diluted with EtOAc (15 mL) and washed with a saturated solution of K_2_CO_3_ (3 × 15 mL). The combined organic layers were dried over MgSO_4_, filtered and the solvents were removed *in vacuo* to yield **3a**–**o** [46] without the need of further purification.

*1-(4-Nitrophenyl)but-3-en-1-ol* (**3a**): Yield: 181 mg (94%); ^1^H-NMR (CDCl_3_) δ 8.20 (d, *J* = 8.7 Hz, 2H, H_Aryl_), 7.53 (d, *J* = 8.7 Hz, 2H, H_Aryl_), 5.85–5.71 (m, 1H, C*H*=CH_2_), 5.22–5.16 (m, 2H, CH=C*H*_2_), 4.86 (dd, *J* = 7.8, 4.5 Hz, 1H, C*H*OH), 2.61–2.39 (m, 2H, CHC*H*_2_), 2.15 (br s, 1H, OH); ^13^C-NMR (CDCl_3_) δ 151.1, 147.1, 133.1, 126.5, 123.5, 119.5, 72.1, 43.8.

*1-(3-Nitrophenyl)but-3-en-1-ol* (**3b**): Yield: 154 mg (82%); ^1^H-NMR (CDCl_3_) δ 8.23 (t, *J* = 1.5 Hz, 1H, H_Aryl_), 8.12 (ddd, *J* = 8.1, 2.1, 0,9 Hz, 1H, H_Aryl_), 7.69 (d, *J* = 8.1 Hz, 1H, H_Aryl_), 7.53 (t, *J* = 8.1 Hz, 1H, H_Aryl_), 5.86–5.72 (m, 1H, C*H*=CH_2_), 5.21–5.15 (m, 2H, CH=C*H*_2_), 4.86 (dd, *J* = 8.1, 5.1 Hz, 1H, C*H*OH), 2.62–2.42 (m, 2H, CHC*H*_2_), 2.17 (br s, 1H, OH); ^13^C-NMR (CDCl_3_) δ 148.1, 145.9, 133.2, 131.9, 129.3, 122.4, 120.8, 119.6, 72.0, 43.9.

*1-(2-Nitrophenyl)but-3-en-1-ol* (**3c**): Yield: 158 mg (80%); ^1^H-NMR (CDCl_3_) δ 7.92 (dd, *J* = 8.1, 1.2 Hz, 1H, H_Aryl_), 7.83 (dd, *J* = 8.1, 1.5 Hz, 1H, H_Aryl_), 7.67 (td, *J* = 8.1, 1.2 Hz, 1H, H_Aryl_), 7.42 (td, *J* = 8.1, 1.2 Hz, 1H, H_Aryl_), 5.96–5.82 (m, 1H, C*H*=CH_2_), 5.31 (dd, *J* = 8.4, 3.6 Hz, 1H, C*H*OH), 5.23–5.17 (m, 2H, CH=C*H*_2_), 2.75–2.66 (m, 1H, CHC*H*_2_), 2.47–2.36 (m, 2H, CHC*H*_2_ and O*H*); ^13^C-NMR (CDCl_3_) δ 147.7, 139.2, 133.9, 133.4, 128.1, 128.0, 124.3, 119.0, 68.3, 42.8.

*1-(4-Fluorophenyl)but-3-en-1-ol* (**3d**): Yield: 144 mg (87%); ^1^H-NMR (CDCl_3_) δ 7.28–7.24 (m, 2H, H_Aryl_), 6.96 (t, 2H, *J* = 8.4 Hz, H_Aryl_), 5.79–5.65 (m, 1H, C*H*=CH_2_), 5.12–5.07 (m, 2H, CH=C*H*_2_), 4.66 (dd, 1H, *J* = 7.2, 5.7 Hz, C*H*OH), 2.50–2.38 (m, 2H, CHC*H*_2_), 1.85 (br s, 1H, OH); ^13^C-NMR δ (CDCl_3_) δ 164.1, 139.2, 133.8, 127.1, 118.4, 114.9, 72.3, 43.6.

*1-(4-Chlorophenyl)but-3-en-1-ol* (**3e**): Yield: 155 mg (85%); ^1^H-NMR (CDCl_3_) δ 7.27–7.19 (m, 4H, H_Aryl_), 5.79–5.62 (m, 1H, C*H*=CH_2_), 5.13–5.05 (m, 2H, CH=C*H*_2_), 4.65 (dd, *J* = 7.5, 5.4 Hz, C*H*OH), 2.48–2.32 (m, 2H, CHC*H*_2_), 2.04 (br s, 1H, OH); ^13^C-NMR (CDCl_3_) δ 142.2, 133.9, 131.1, 128.5, 127.2, 118.8, 72.5, 43.8.

*1-(4-Bromophenyl)but-3-en-1-ol* (**3f**): Yield: 194 mg (86%); ^1^H-NMR (CDCl_3_) δ 7.41 (d, *J* = 8.7 Hz, 2H, H_Aryl_), 7.17 (d, *J* = 8.7 Hz, 2H, H_Aryl_), 5.78–5.64 (m, 1H, C*H*=CH_2_), 5.13–5.06 (m, 2H, CH=C*H*_2_), 4.64 (dd, *J* = 7.8, 5.4 Hz, C*H*OH), 2.49–2.32 (m, 2H, CHC*H*_2_), 1.99 (br s, 1H, OH); ^13^C-NMR (CDCl_3_) δ 142.8, 133.9, 131.4, 127.5, 121.2, 118.9, 72.5, 43.8.

*Non-1-en-4-ol* (**3g**): Yield: 126 mg (89%); ^1^H-NMR (CDCl_3_) δ 5.89-5.76 (m, 1H, C*H*=CH_2_), 5.29–5.09 (m, 2H, CH=C*H*_2_), 3.68–3.59 (m, 1H, C*H*OH), 2.34–2.25 (m, 2H, CHC*H*_2_), 2.18–2.07 (m, 2H, C*H*OH) 1.66 (br s, 1H, OH), 150–1.25 (m, 6H, CH_3_C*H*_2_C*H*_2_C*H*_2_), 0.87 (t, *J* = 6.3 Hz, 6H, C*H*_3_CH_2_); ^13^C-NMR (CDCl_3_) δ 134.9, 118.0, 70.6, 41.9, 36.7, 31.8, 25.3, 22.6, 14.0.

*(E)-1-Phenylhexa-1,5-dien-3-ol* (**3h**): Yield: 157 mg (90%); ^1^H-NMR (CDCl_3_) δ 7.40–7.21 (m, 5H, H_Aryl_), 6.61 (dd, *J* = 15.9, 1.2 Hz, 1H, PhC*H=*CH), 6.24 (dd, *J* = 15.9, 6.3 Hz, 1H, PhCH*=*C*H*), 5.86 (ddt, *J* = 17.1, 10.2, 6.9 Hz, 1H, C*H*=CH_2_), 5.22–5.14 (m, 2H, CH=C*H*_2_), 4.39–4.33 (m, 1H, C*H*OH), 2.50–2.34 (m, 2H, CHC*H*_2_), 1.78 (br s, 1H, OH); ^13^C-NMR (CDCl_3_) δ 136.5, 133.9, 131.5, 130.2, 128.5, 127.6, 126.4, 118.3, 71.6, 41.9.

*1-(Naphthalen-2-yl)but-3-en-1-ol* (**3i**): Yield: 172 mg (87%); ^1^H-NMR (CDCl_3_) δ 7.86–7.81 (m, 4H, H_Aryl_), 7.53–7.47 (m, 3H, H_Aryl_), 5.84 (ddt, *J* = 17.1, 10.2, 7.5 Hz, 1H, C*H*=CH_2_), 5.23–5.14 (m, 2H, CH=C*H*_2_), 4.91 (dd, *J* = 7.2, 5.1 Hz, 1H, C*H*OH), 2.61–2.57 (m, 2H, CHC*H*_2_), 2.10 (br s, 1H, OH); ^13^C-NMR (CDCl_3_) δ 141.2, 134.3, 133.2, 132.9, 128.1, 127.9, 127.6, 126.1, 125.8, 124.2, 123.9, 118.4, 73.3, 43.6.

*1-Phenyl-but-3-en-1-ol* (**3j**): Yield: 130 mg (88%); ^1^H-NMR (CDCl_3_) δ 7.36–7.24 (m, 5H, H_Aryl_), 5.87–5.73 (m, 1H, C*H*=CH_2_), 5.19–5.11 (m, 2H, C*H*=CH_2_), 4.73 (dd, *J* = 7.5, 5.4 Hz, 1H, C*H*OH), 2.54–2.47 (m, 2H, CHC*H*_2_), 2.00 (br s, 1H, OH); ^13^C-NMR δ (CDCl_3_) δ 143.8, 134.4, 128.2, 127.3, 125.7, 118.0, 73.2, 43.6.

*1-p-Tolyl-but-3-en-1-ol* (**3k**): Yield: 133 mg (82%); ^1^H-NMR (CDCl_3_) δ 7.22 (d, *J* = 7.8 Hz, 2H, H_Aryl_), 7.16 (d, *J* = 7.8 Hz, 2H, H_Aryl_), 5.81 (ddd, 1H, *J* = 17.1, 10.2, 6.6 Hz, C*H*=CH_2_), 5.20–5.11 (m, 2H, CH=C*H*_2_), 4.71 (t, *J* = 6.6 Hz, C*H*OH), 2.56–2.48 (m, 2H, CHC*H*_2_), 2.35 (s, 3H, CH_3_), 1.78 (br s, 1H, OH); ^13^C-NMR (CDCl_3_) δ 140.9, 137.2, 134.6, 129.0, 125.7, 118.2, 73.1, 43.7, 21.1.

*1-(4-Methoxyphenyl)but-3-en-1-ol* (**3l**): Yield: 158 mg (89%); ^1^H-NMR (CDCl_3_) δ 7.77 (d, *J* = 9.0 Hz, 2H, H_Aryl_), 7.37 (d, *J* = 9.0 Hz, 2H, H_Aryl_), 6.29 (ddt, *J* = 16.8, 9.9, 6.6 Hz, 1H, C*H*=CH_2_), 5.68–5.59 (m, 2H, CH=C*H*_2_), 5.17 (t, *J* = 6.6 Hz, C*H*OH), 4.29 (s, 3H, OMe), 3.01–2.96 (m, 2H, CHC*H*_2_), 2.50 (br s, 1H, OH); ^13^C-NMR (CDCl_3_) δ 158.9, 136.0, 134.6, 127.0, 118.2, 113.7, 72.9, 55.2, 43.7.

*1-(3-Methoxyphenyl)but-3-en-1-ol* (**3m**): Yield: 160 mg (90%); ^1^H-NMR (CDCl_3_) δ 7.29 (dd, *J* = 8.1, 7.8 Hz, 1H, H_Aryl_), 6.97–6.94 (m, *2*H, H_Aryl_), 6.84 (ddd, *J* = 8.1, 2.7, 1.2 Hz, 1H, H_Aryl_), 5.84 (ddt, *J* = 17.1, 10.2, 7.5 Hz, 1H, C*H*=CH_2_), 5.23–5.15 (m, 2H, CH=C*H*_2_), 4.74 (dd, *J* = 7.5, 5.4 Hz, 1H, C*H*OH), 3.84 (s, 3H, OMe), 2.56–2.50 (m, 2H, CHC*H*_2_), 2.06 (br s, 1H, OH); ^13^C-NMR (CDCl_3_) δ 159.6, 145.6, 134.4, 129.3, 118.2, 118.0, 112.9, 111.2, 73.1, 55.1, 43.6. 

*1-(2-Methoxyphenyl)but-3-en-1-ol* (**3n**): Yield: 162 mg (91%); ^1^H-NMR (CDCl_3_) δ 7.33 (dd, *J* = 7.5, 1.8 Hz, 1H, H_Aryl_), 7.28 (td, *J* = 7.5, 1.8 Hz, 1H, H_Aryl_), 6.95 (td, *J* = 8.4, 1.2 Hz, 1H, H_Aryl_), 6.87 (d, *J* = 8.4 Hz, 1H, H_Aryl_), 5.85 (ddt, *J* = 17.1, 10.2, 7.5 Hz, 1H, C*H*=CH_2_), 5.17–5.08 (m, 2H, CH=C*H*_2_), 4.95 (dd, *J* = 8.1, 5.1 Hz, 1H, C*H*OH), 3.84 (s, 3H, OMe), 2.64–2.44 (m, 2H, CHC*H*_2_), 2.41 (br s, 1H, OH); ^13^C-NMR (CDCl_3_) δ 156.2, 135.1, 131.7, 128.2, 126.7, 120.6, 117.4, 110.3, 69.5, 55.1, 41.8.

*1-(Furan-2-ylbut-3-en-1-ol* (**3o**): Yield: 119 mg (86%); ^1^H-NMR (CDCl_3_) δ 7.38 (dd, *J* = 1.8, 0.9 Hz, 1H, H_Het_), 6.33 (dd, *J* = 2.1, 1.8 Hz, 1H, H_Het_), 6.25 (dd, *J* = 2.1, 0.9 Hz, 1H, H_Het_), 5.81 (ddt, *J* = 17.1, 10.2, 6.9 Hz, 1H, C*H*=CH_2_), 5.23–5.13 (m, 2H, CH=C*H*_2_), 4.75 (dd, *J* = 6.6, 6.3 Hz, 1H, C*H*OH), 2.66–2.60 (m, 2H, CHC*H*_2_), 2.15 (br s, 1H, OH); ^13^C-NMR (CDCl_3_) δ 155.6; 141.9; 133.6, 118.6, 110.1, 106.1, 66.9, 40.1. 

### 3.3. General Procedure for the Crotylation of ***1a*** with Potassium (E)-crotyltrifluoroborate ***(4)*** Catalyzed by 18-C-6

To a solution of aldehyde **1a** (38 mg, 0.25 mmol) in CH_2_Cl_2_ (1 mL) was added 18-*C*-6 (6.5 mg, 10 mol%) followed by potassium (*E*)-crotyltrifluoroborate (**4**, 49 mg, 0.3 mmol) and water (1 mL). The biphasic mixture was stirred for 15 min, diluted with EtOAc (15 mL) and washed with a saturated solution of K_2_CO_3_ (3 × 10 mL). The combined organic layers were dried over MgSO_4_, filtered and the solvents were removed *in vacuo* to yield **5** [47] without the need of further purification.

*2-Methyl-1-(4-nitrophenyl)but-3-en-1-ol* (**5**): Yield: 55 mg (89%); ^1^H-NMR (CDCl_3_) δ 8.21 (d, *J* = 8.7 Hz, 2H, H_Aryl_), 7.51 (d, *J* = 8.7 Hz, 2H, H_Aryl_), 5.75 (ddd, *J* = 16.8, 10.5, 8.1 Hz, 1H, C*H*=CH_2_), 5.23–5.15 (m, 2H, CH=C*H*_2_), 4.52 (d, *J* = 7.2 Hz, 1H, C*H*OH), 2.47 (sex, *J* = 7.2 Hz, 2H, CHC*H*), 2.17 (br s, 1H, OH), 0.94 (d, *J* = 7.2 Hz, 3H, CH_3_); ^13^C-NMR (CDCl_3_) δ 15.0, 139.4, 127.8, 123.4, 118.1, 76.9, 46.5, 25.0, 16.5.

### 3.4. General Procedure for the Allylation of Compound 6 with Potassium Allyltrifluoroborate ***(2)*** Catalyzed by 18-C-6

To a solution of **6** (150 mg, 0.5 mmol) in CH_2_Cl_2_ (1.5 mL) was added 18-*C*-6 (13 mg, 10 mol%) followed by potassium allyltrifluoroborate (**2**, 89 mg, 0.60 mmol) and water (1.5 mL). The biphasic mixture was stirred for 15 minutes, diluted with EtOAc (15 mL) and washed with a saturated solution of K_2_CO_3_ (3 x 15 mL). The combined organic layers were dried over MgSO_4_, filtered and the solvents were removed *in vacuo* to yield **7** (138 mg, 85%) without the need of further purification.

*(5S,7Z,9E)-5-(Methoxymethoxy)-11-(tetrahydro-2H-pyran-2-yloxy)undeca-1,7,9-trien-3-ol*: ^1^H-NMR (CDCl_3_) δ 6.57–6.47 (m, 1H), 6.11 (td, *J* = 11.1, 1.2 Hz, 1H), 5.90–5.75 (m, 2H), 5.44 (dd, *J* = 18.6, 7.5 Hz, 1H), 5.14–5.07 (m, 3H), 4.73 (dd, *J* = 15.6, 7.5 Hz, 2H), 4.66–4.63 (m, 2H), 4.30 (dd; *J* = 12.9, 6.0 Hz, 1H), 4.03 (dd, *J* = 12.9, 6.5 Hz, 1H), 3.95-3.78 (m, 3H), 3.55–3.46 (m, 1H), 3.39 (s, 3H), 2.50–2.41 (m, 1H), 2.23 (t, *J* = 6.6 Hz, 3H) 1.88–1.48 (m, 7H); ^13^C-NMR (CDCl_3_) δ 134.89, 130.25, 127.57, 127.22, 126.81, 117.53, 97.88, 96.32, 95.22, 75.31, 67.38, 67;09, 62.15, 55.79, 41.98, 40.97, 30.57, 25.40, 19.41.

## 4. Conclusions

In summary, we have demonstrated that a catalytic amount of 18-*C*-6 can efficiently promote the allylation of aldehydes. This green method features the use of small catalyst loads, avoid the preparation of unstable allyl organometallics and the products were obtained in short reaction times with high yield and purity at room temperature. The approach is complementary to the previously described methods for the synthesis of homoallylic alcohols and should be synthetically useful for the synthesis of more complex compounds.

## Supplementary Materials

Supplementary materials can be accessed at: http://www.mdpi.com/1420-3049/17/12/14099/s1.
