# Supplementary material for: Allylation of Functionalized Aldehydes by Potassium Allyltrifluoroborate Catalyzed by 18-Crown-6 in Aqueous Media
Source: Molecules. 2012 Nov 28;17(12):14099–110. doi: 10.3390/molecules171214099 (PMC6268519; doi:10.3390/molecules171214099)

# Supplementary Materials

## General Remarks

$^{1}$H-NMR and $^{13}$C-NMR data were recorded in $CDCl_3$. The chemical shifts are reported as delta (δ) units in parts per million (ppm) relative to the solvent residual peak as the internal reference. Coupling constants (*J*) for all spectra are reported in Hertz (Hz). Reactions were monitored by thin-layer chromatography on 0.25 mm E. Merck silica gel 60 plates (F254) using UV light, vanillin and *p*-anisaldehyde as visualizing agents. Potassium allyltrifluoroborate [S1] and potassium (*E*)-crotyltrifluoroborate [S2] were prepared according to the literature procedures.

## References


S1. Molander, G.A.; Figueroa, R.A. *cis*-Dihydroxylation of unsaturated potassium alkyl- and aryltrifluoroborates. *Org. Lett*. 2006, *8*, 75–78.

S2. Batey, R.A.; Thadani, A.V.; Smil, D.V. Potassium allyl- and crotyltrifluoroborates: Stable and efficient agents for allylation and crotylation. *Tetrahedron Lett*. **1999**, *40*, 4289–4292.

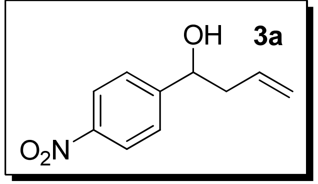


**3a**

**Figure S1.** $^{1}$H-NMR spectrum (300 MHz, $CDCl_3$) of **3a**.

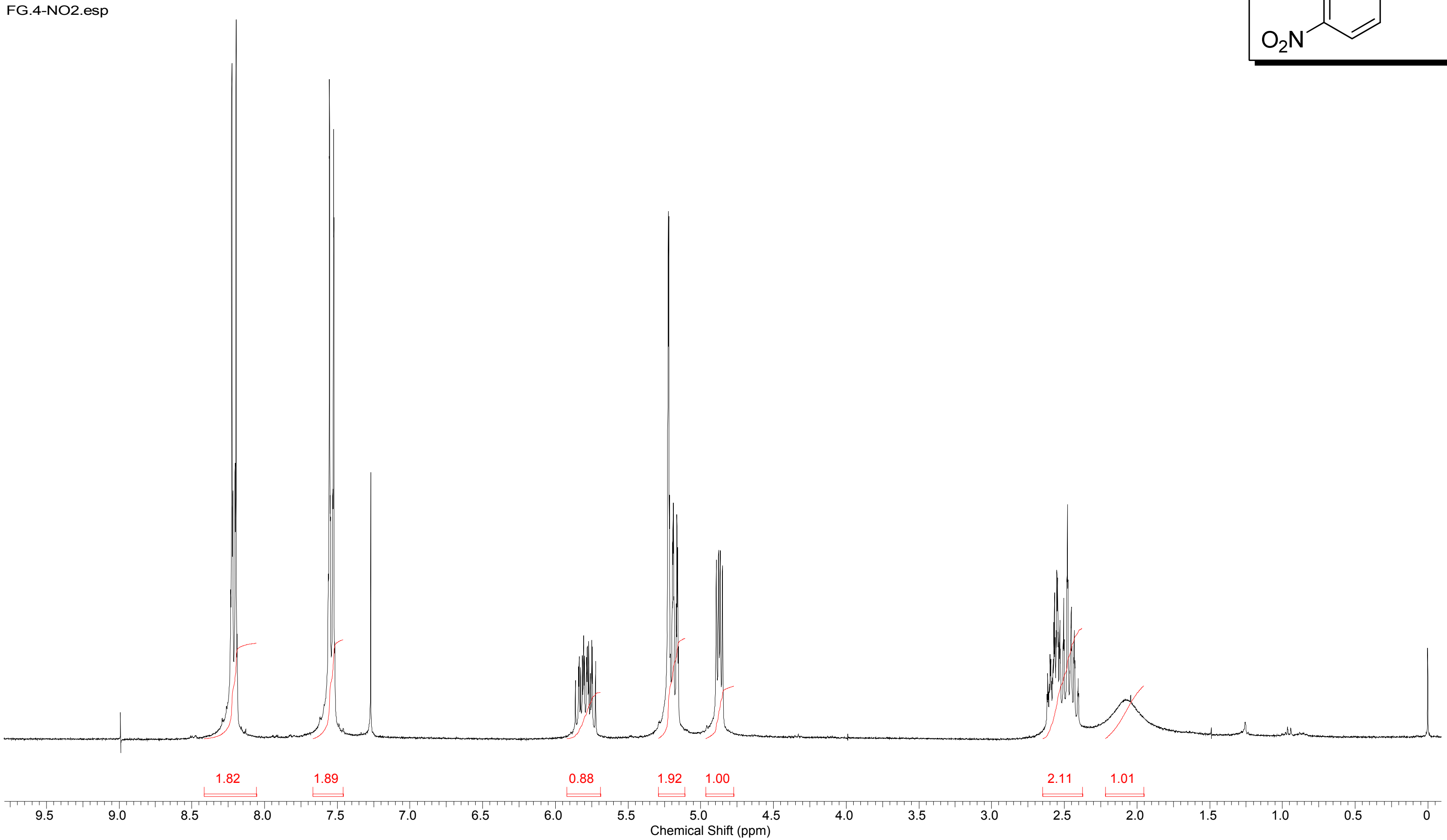

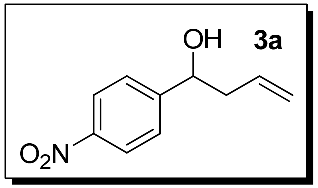


**Figure S2.** $^{13}$C-NMR spectrum (75 MHz, $CDCl_3$) of **3a**.

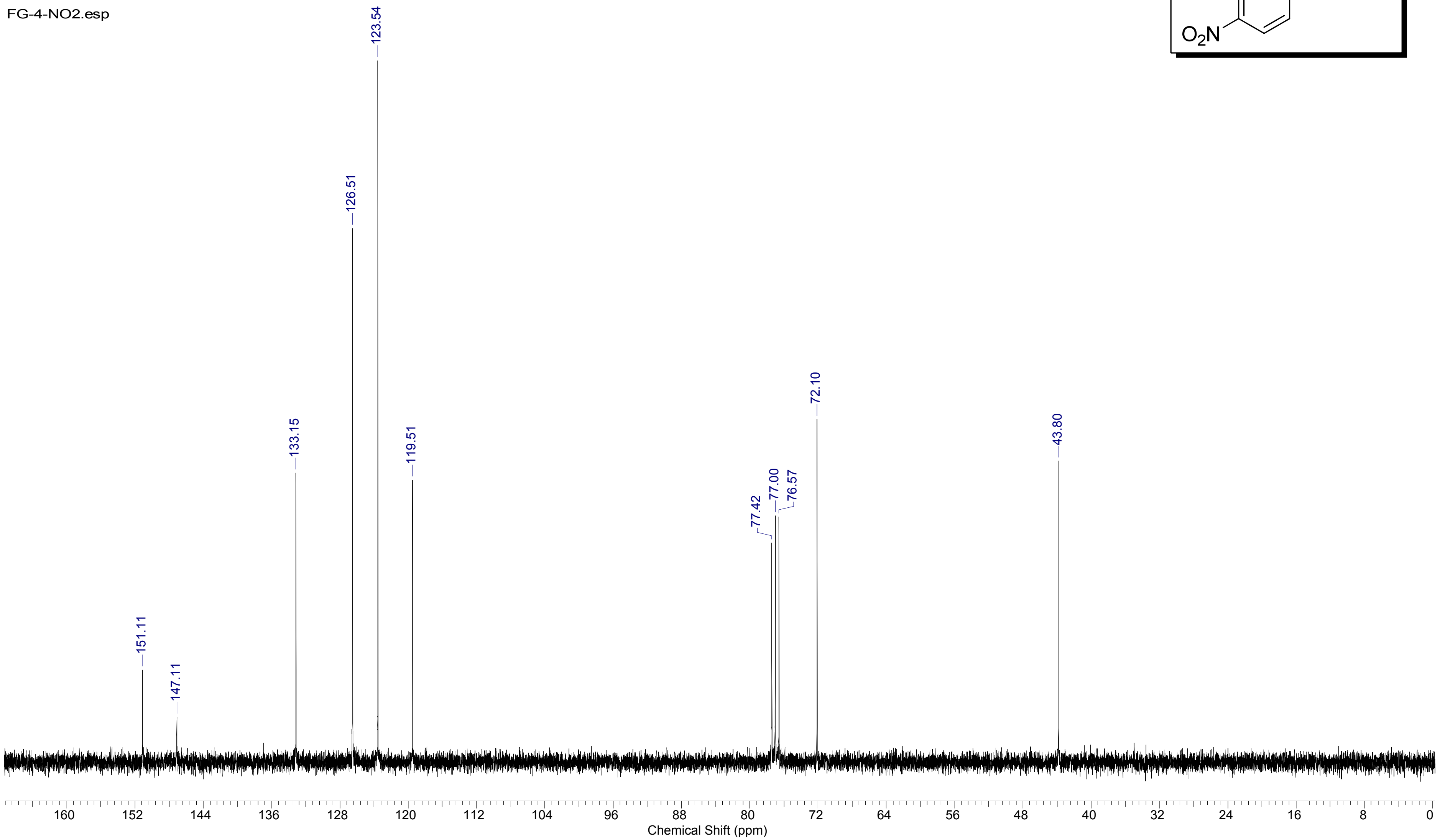

**Figure S3.** $^{1}$H-NMR spectrum (300 MHz, $CDCl_3$) of **3b**.

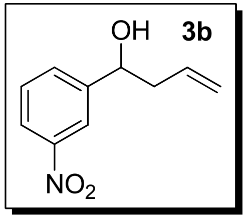


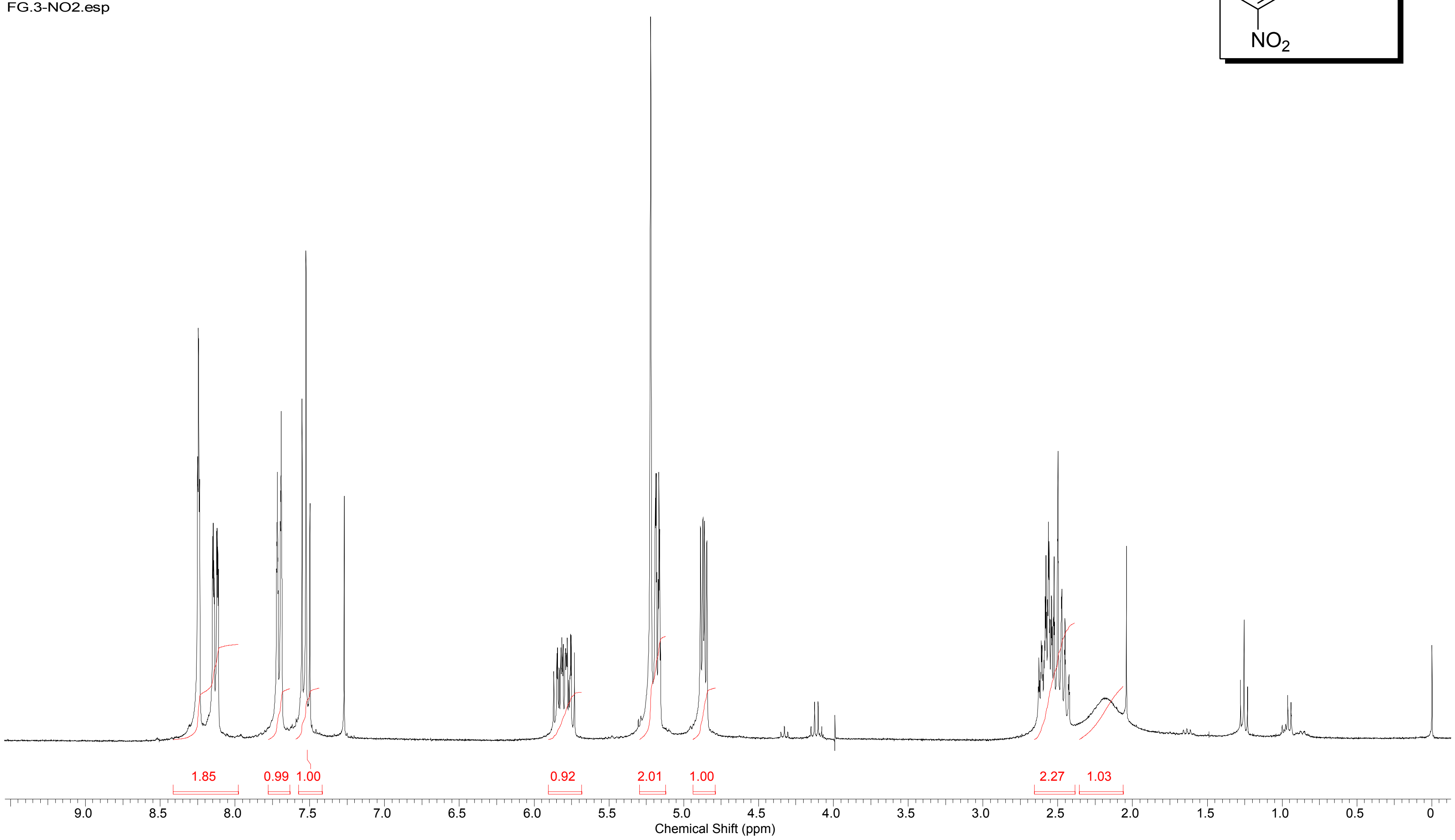

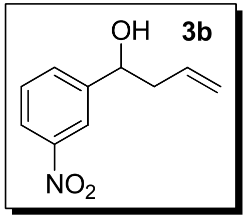


**Figure S4.** $^{13}$C-NMR spectrum (75 MHz, $CDCl_3$) of **3b**.

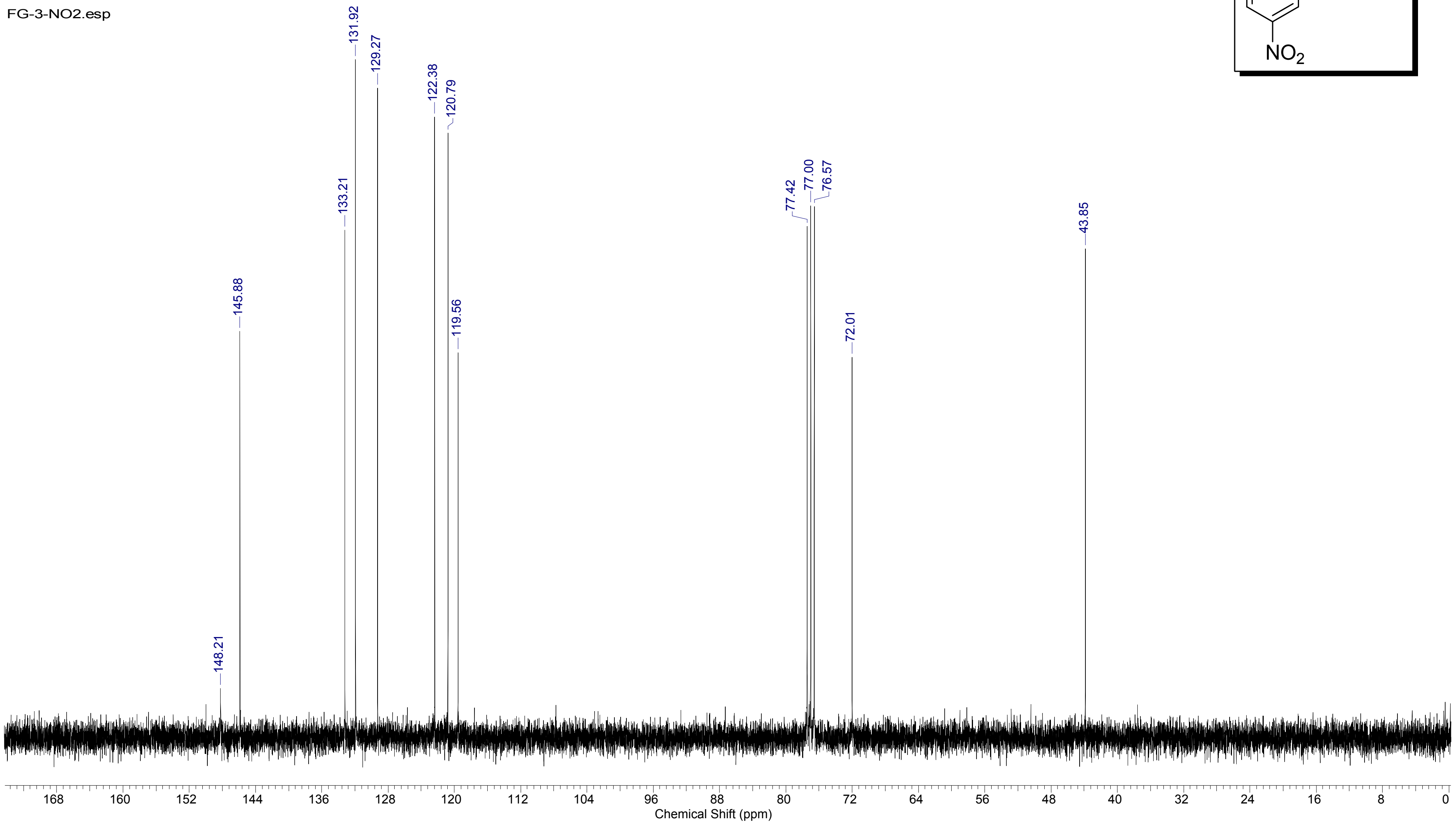

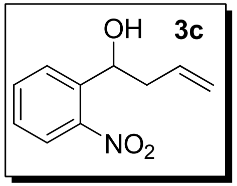

3c

**Figure S5.** ¹H-NMR spectrum (300 MHz, $CDCl_3$) of **3c**.

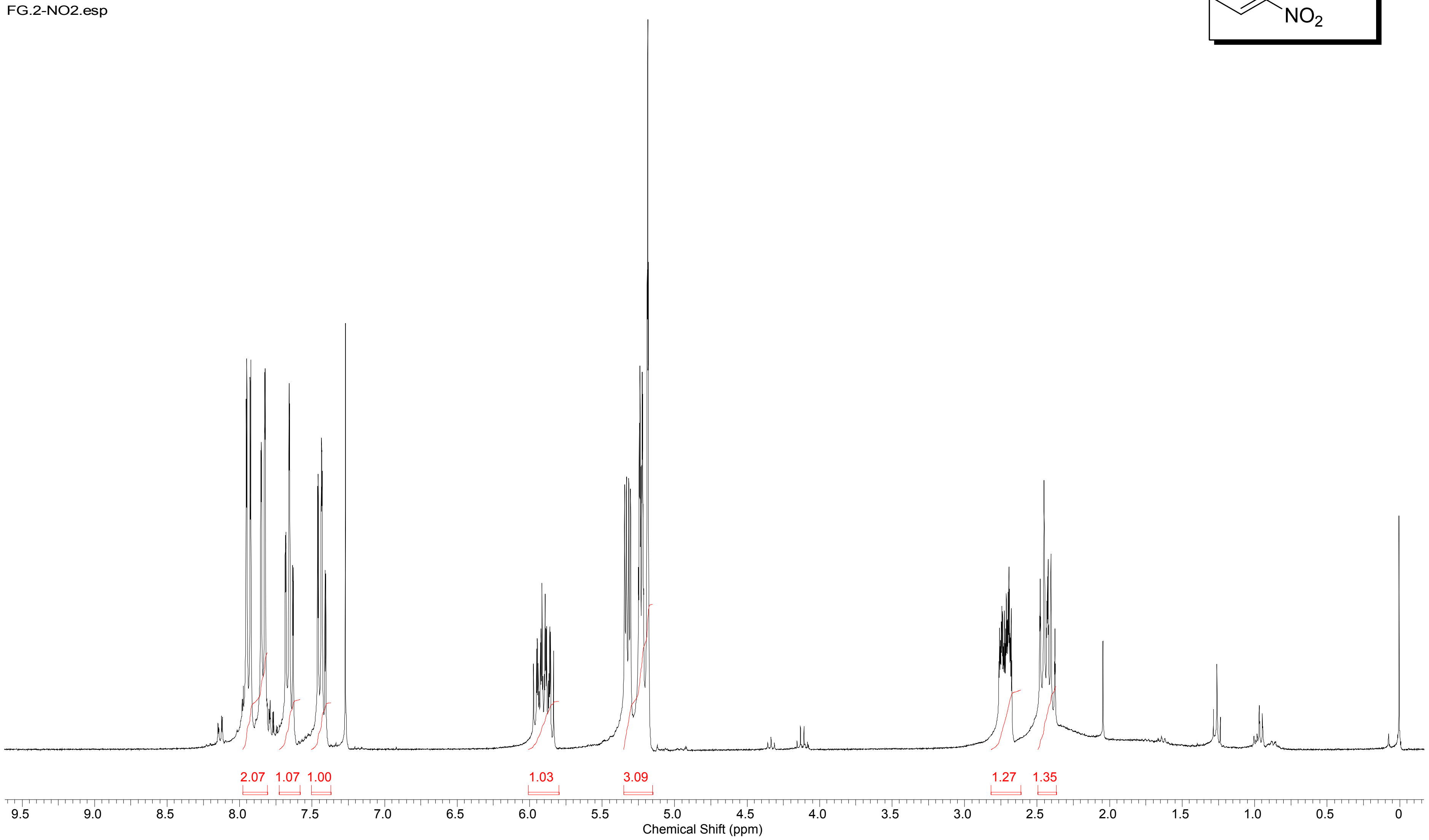

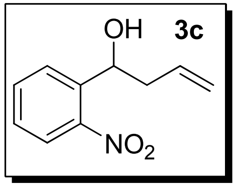


**Figure S6.** $^{13}$C-NMR spectrum (75 MHz, $CDCl_3$) of **3c**.

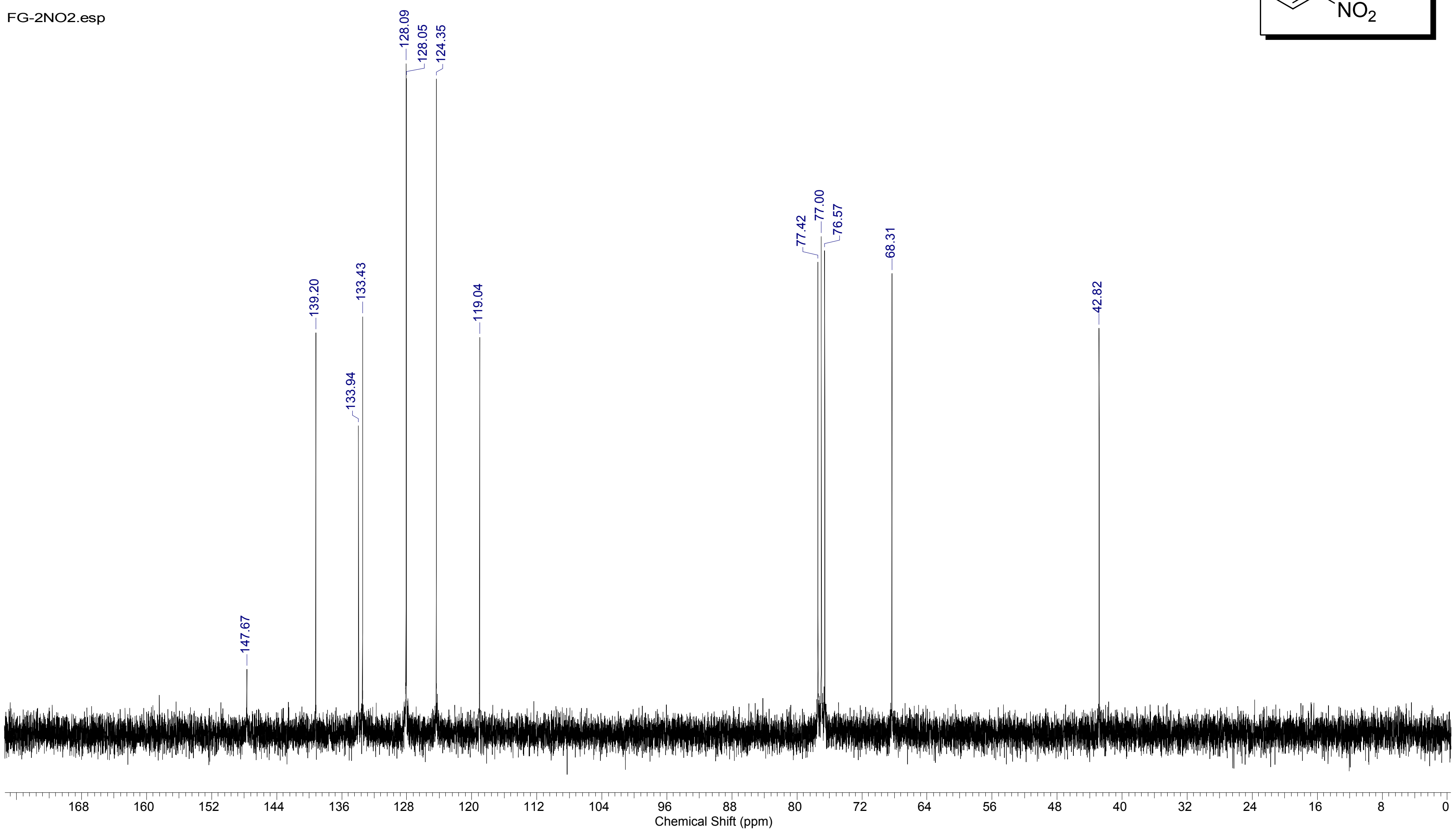

**Figure S7.** $^{1}$H-NMR spectrum (300 MHz, $CDCl_3$) of **3d**.

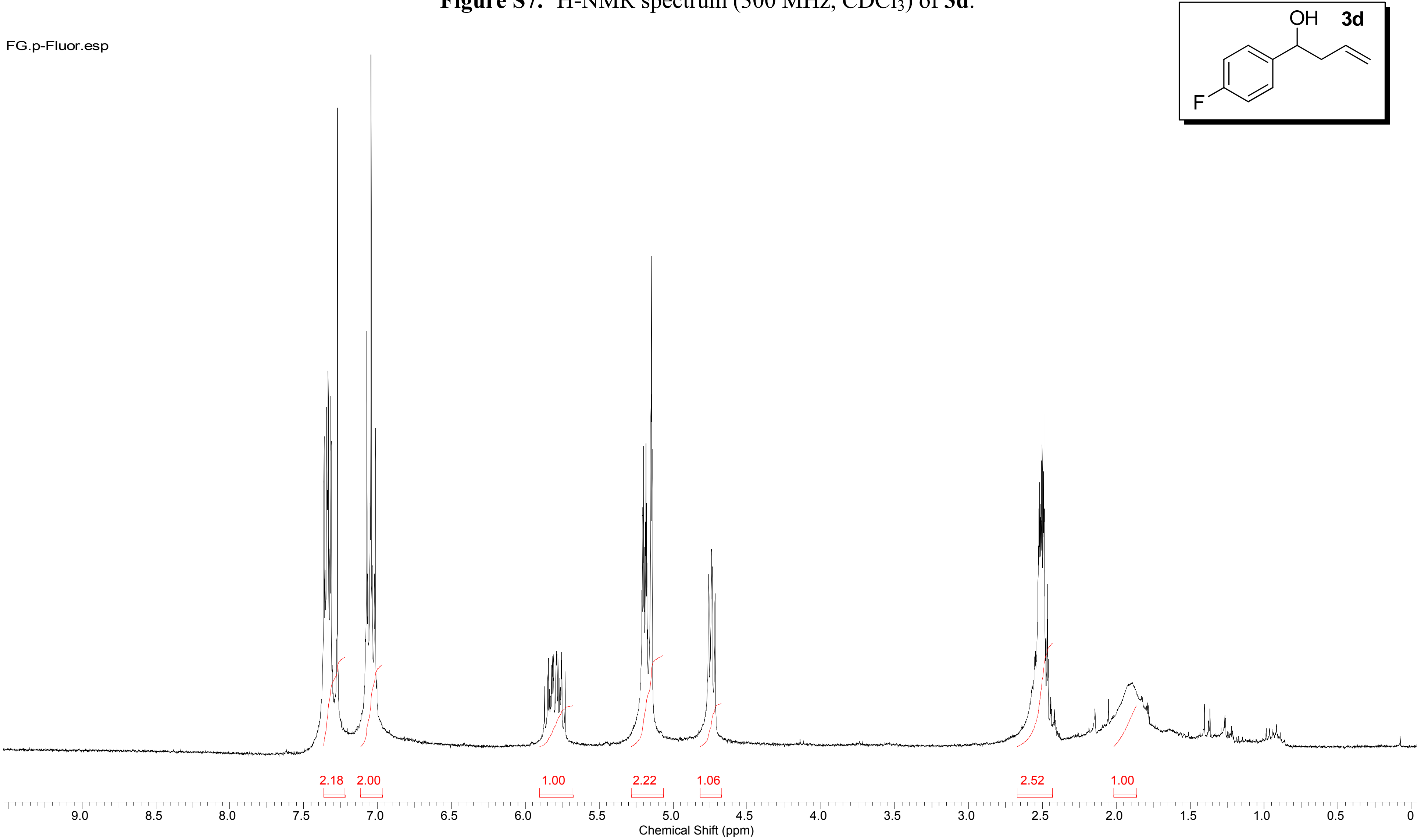

**Figure S8.** $^{13}$C-NMR spectrum (75 MHz, $CDCl_3$) of **3d**.

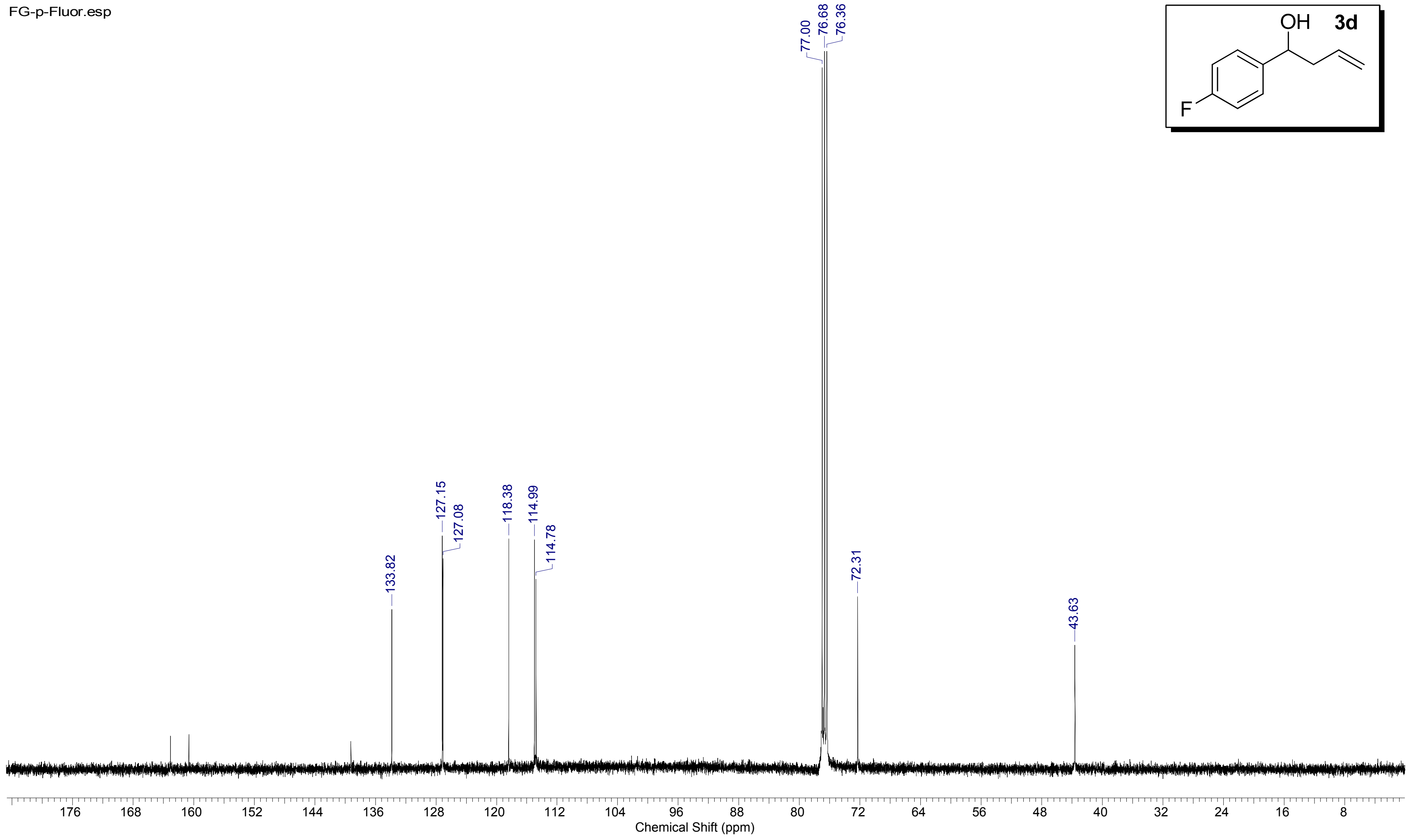

**Figure S9.** $^1$H-NMR spectrum (300 MHz, $CDCl_3$) of **3e**.

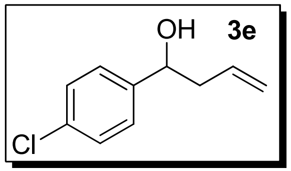


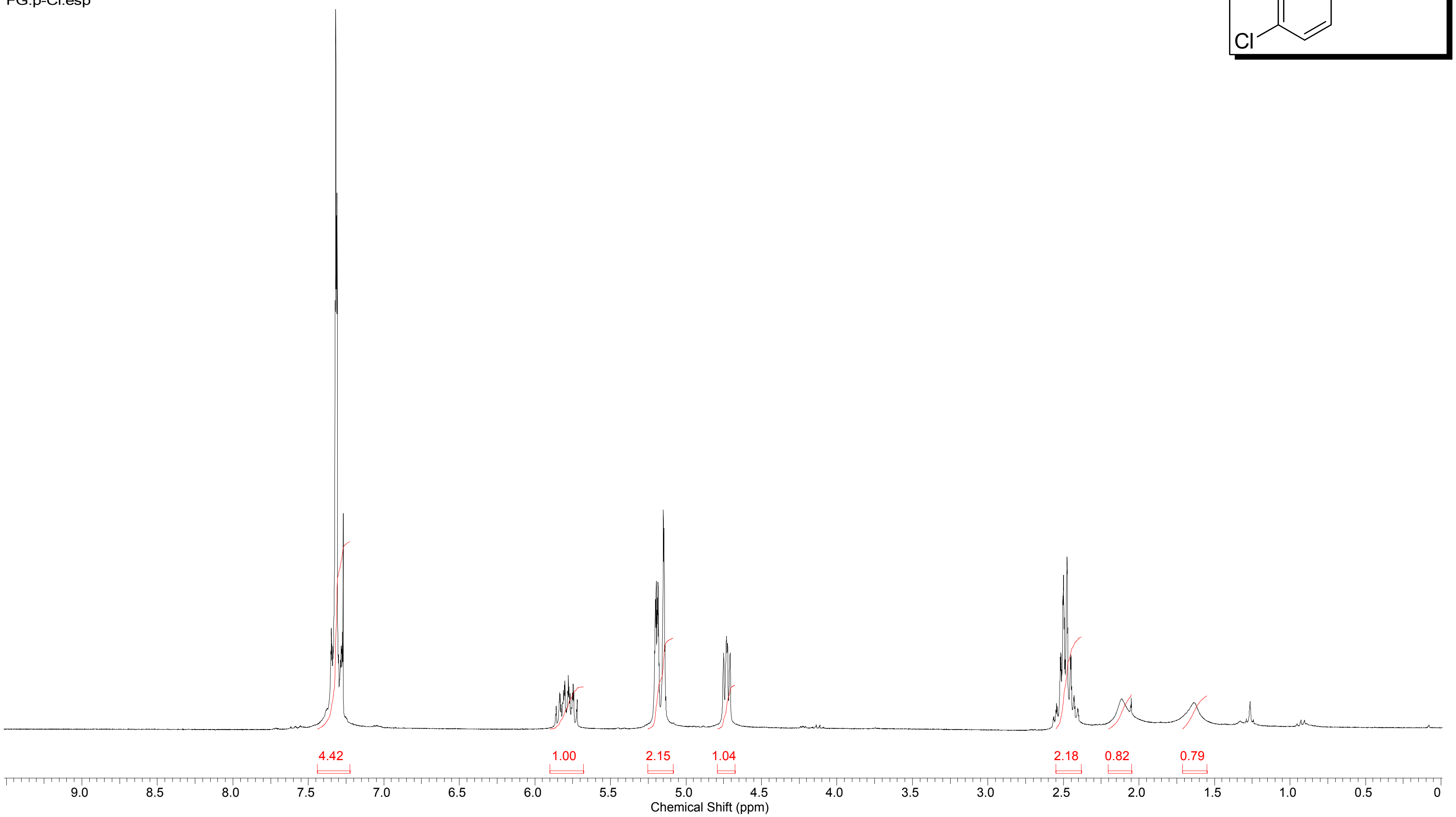

**Figure S10.** $^{13}$C-NMR spectrum (75 MHz, $CDCl_3$) of **3e**.

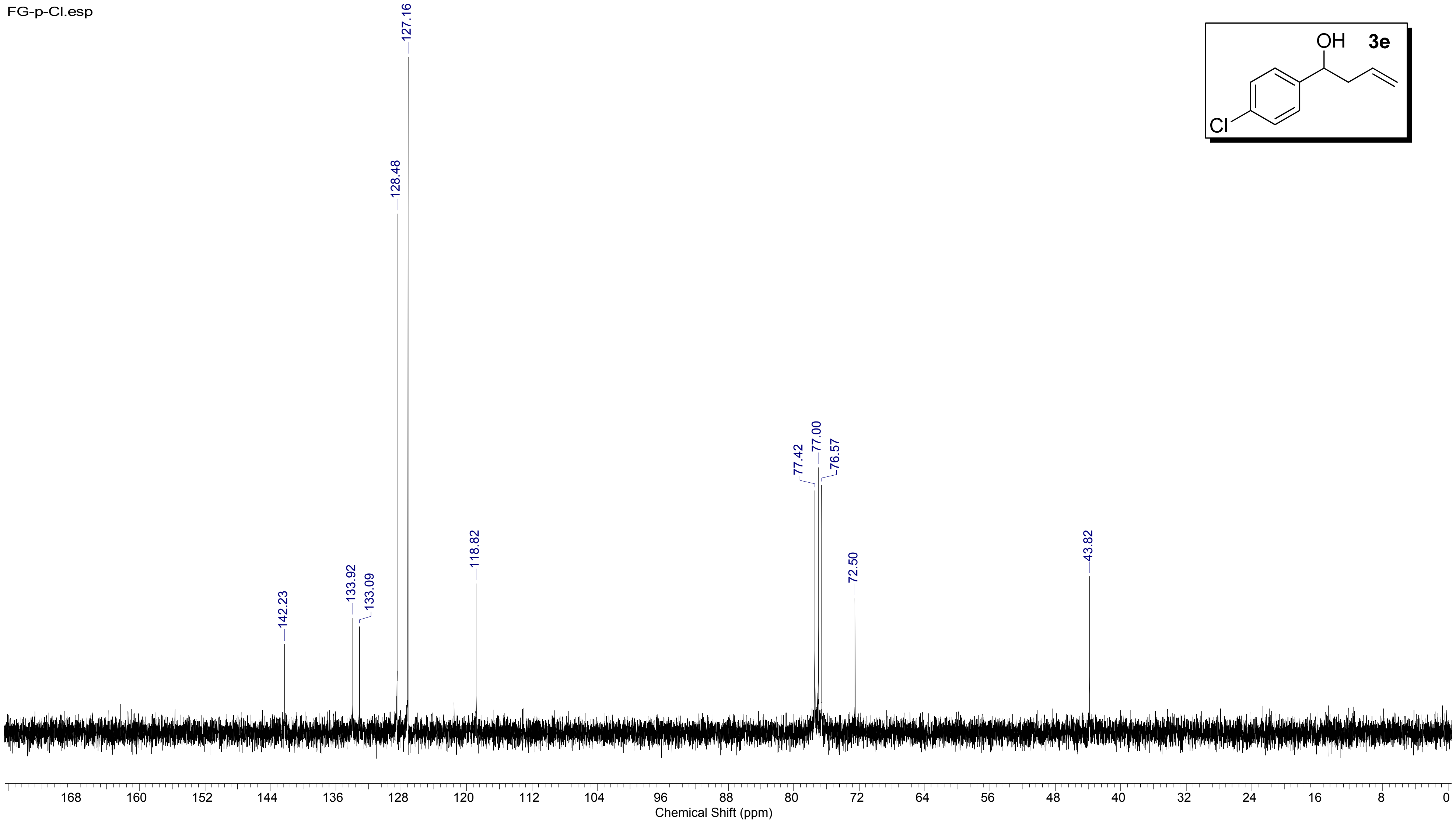

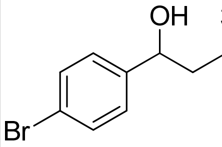


**3f**

**Figure S11.** $^1$H-NMR spectrum (300 MHz, $CDCl_3$) of **3f**.

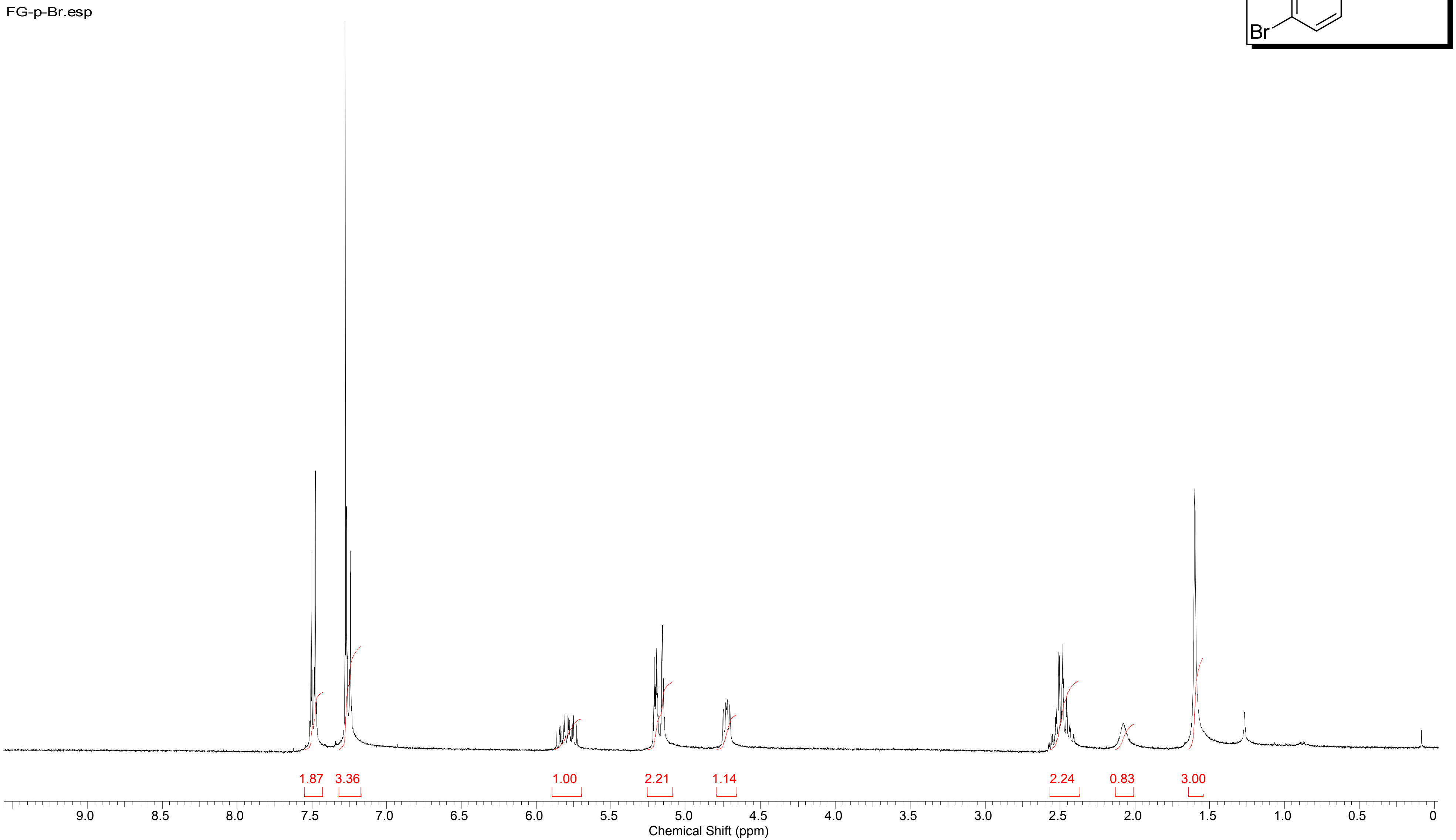

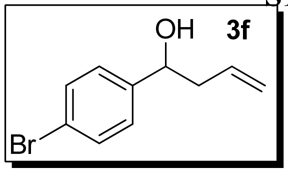


**Figure S12.** $^{13}$C-NMR spectrum (75 MHz, $CDCl_3$) of **3f**.

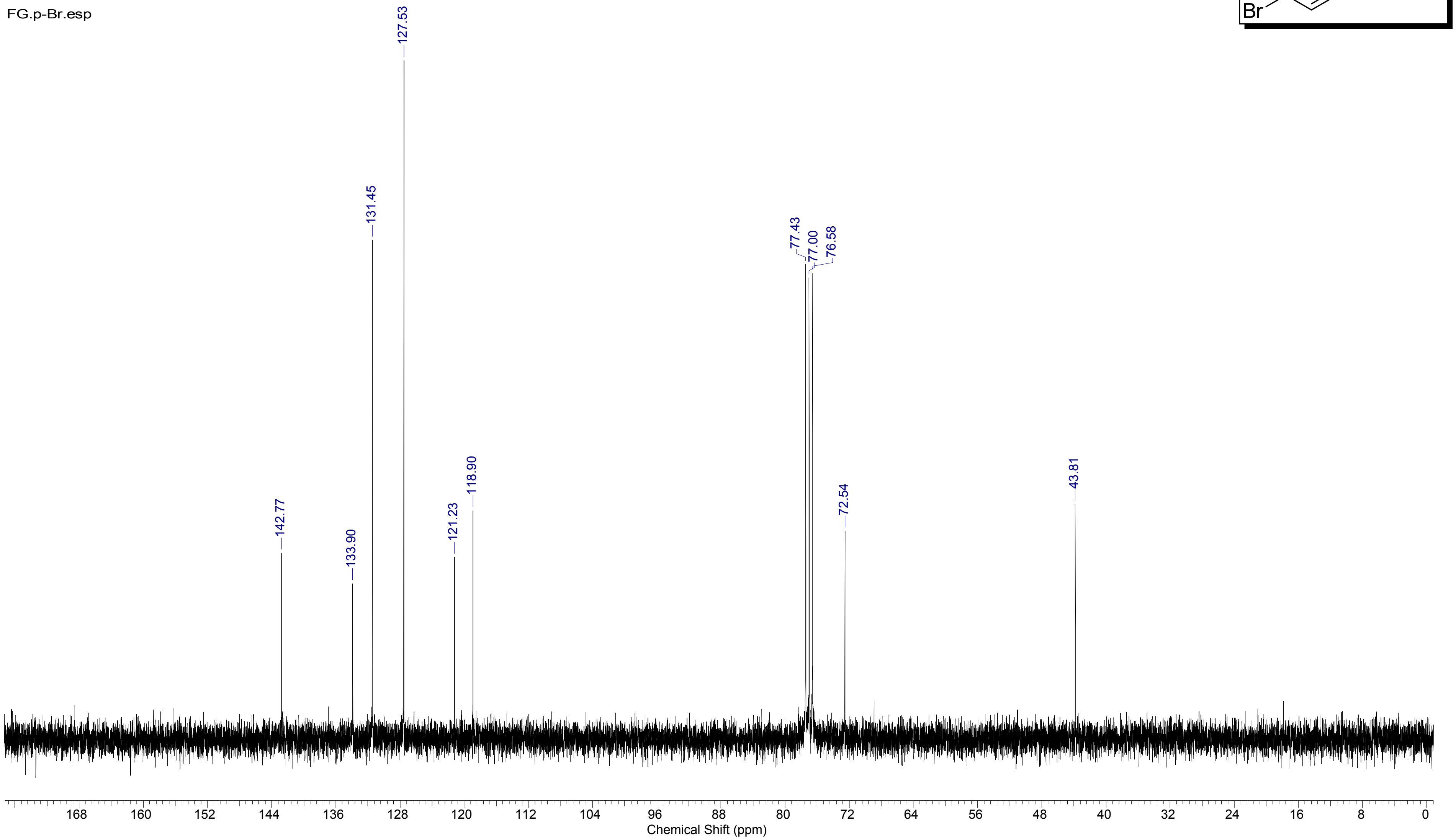

**Figure S13.** $^{1}$H-NMR spectrum (300 MHz, $CDCl_3$) of **3g**.

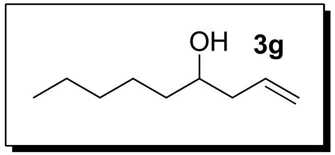


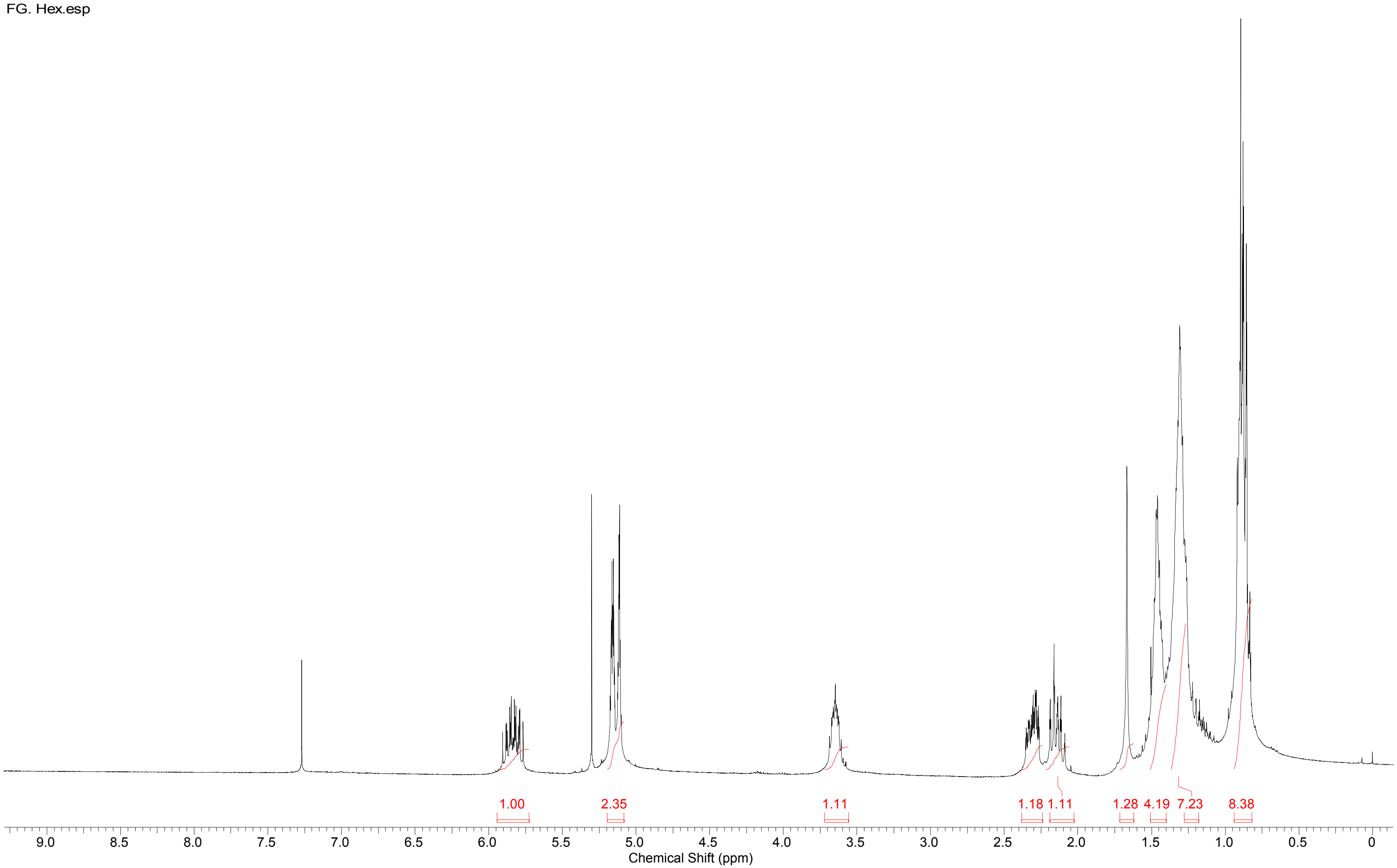

**Figure S14.** $^{13}$C-NMR spectrum (75 MHz, $CDCl_3$) of **3g**.

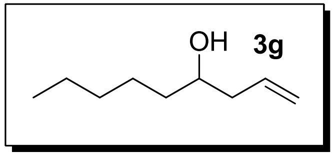


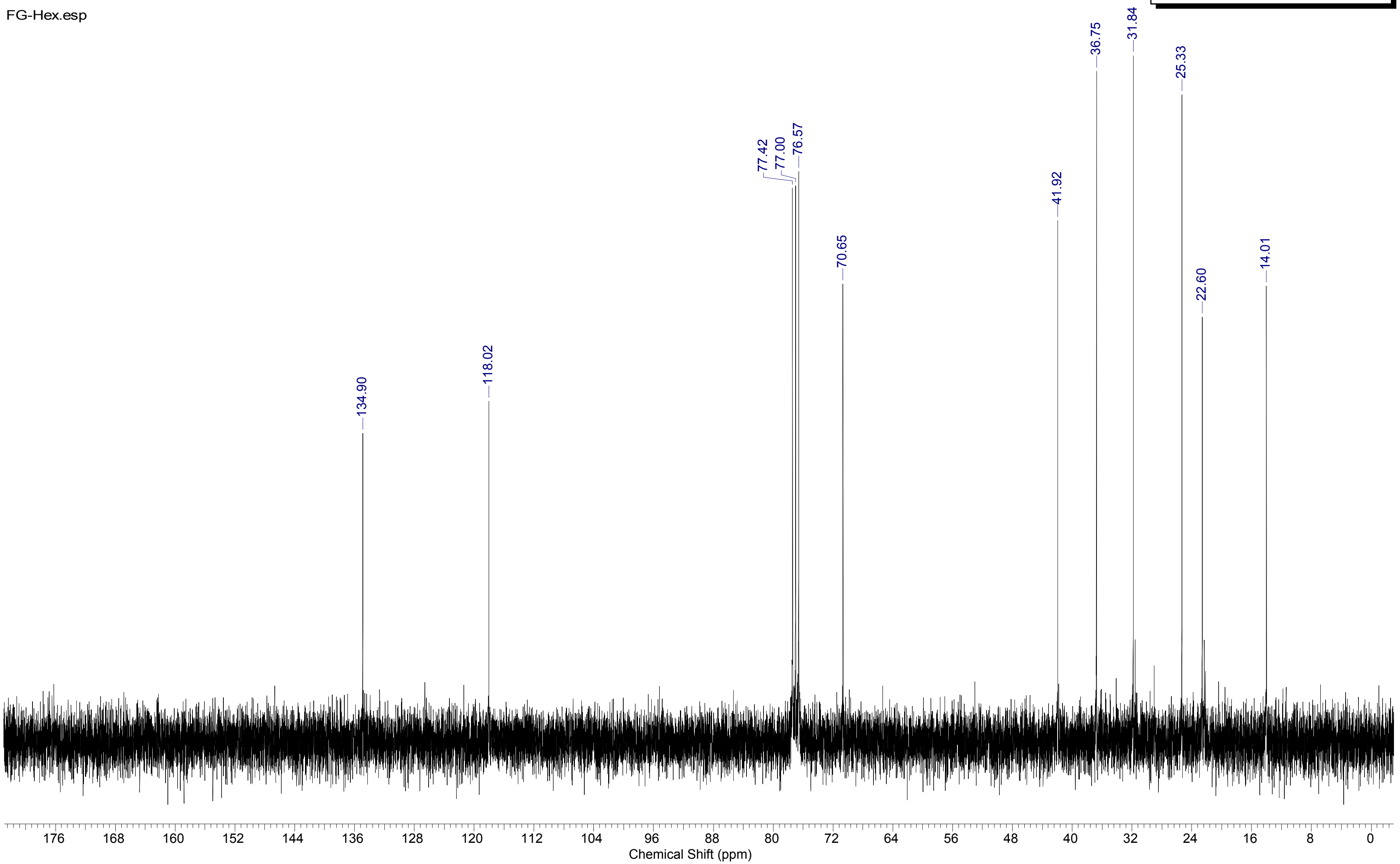

**Figure S15.** $^1$H-NMR spectrum (300 MHz, $CDCl_3$) of **3h**.

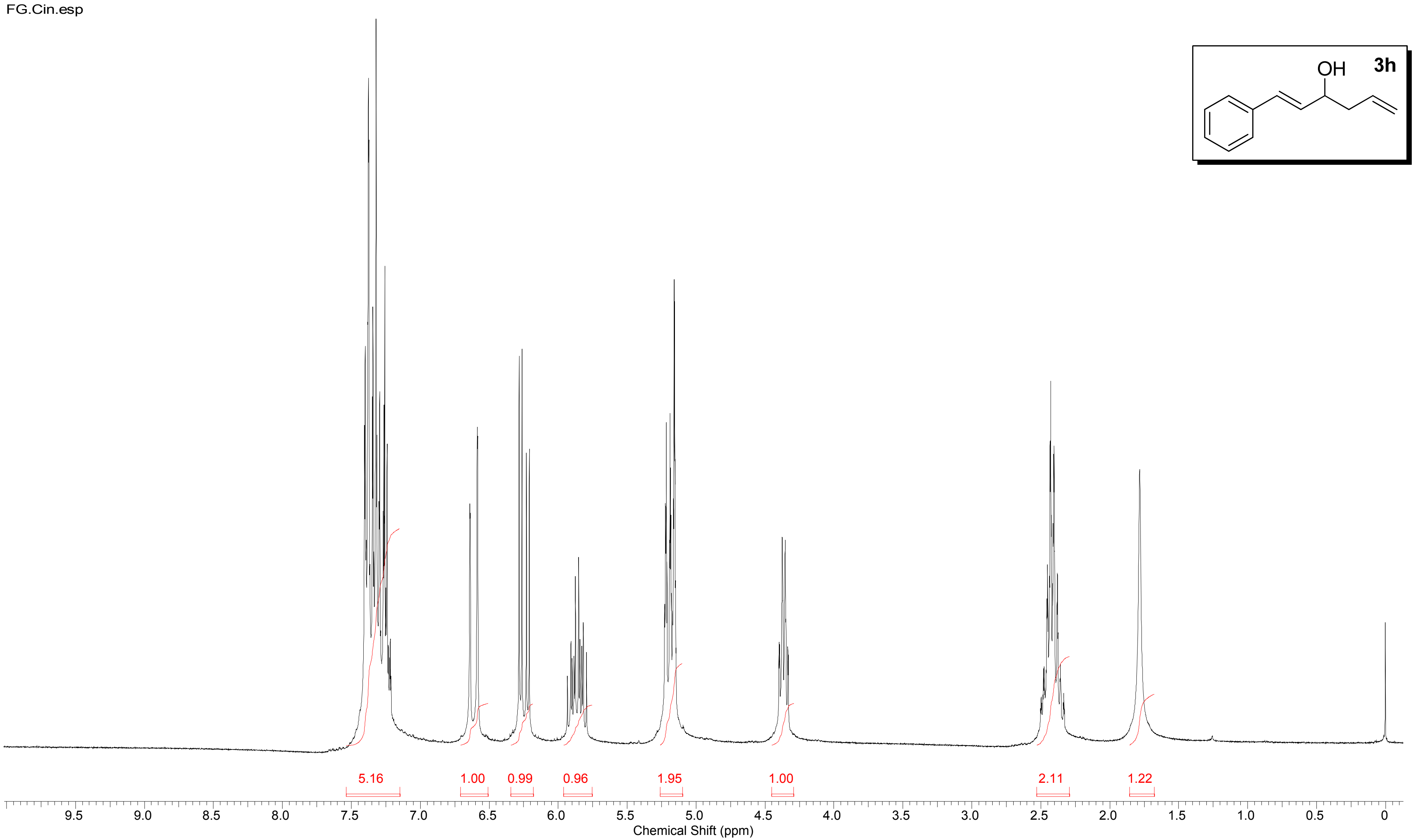

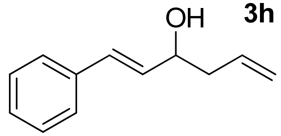

3h

**Figure S16.** $^{13}$C-NMR spectrum (75 MHz, $CDCl_3$) of **3h**.

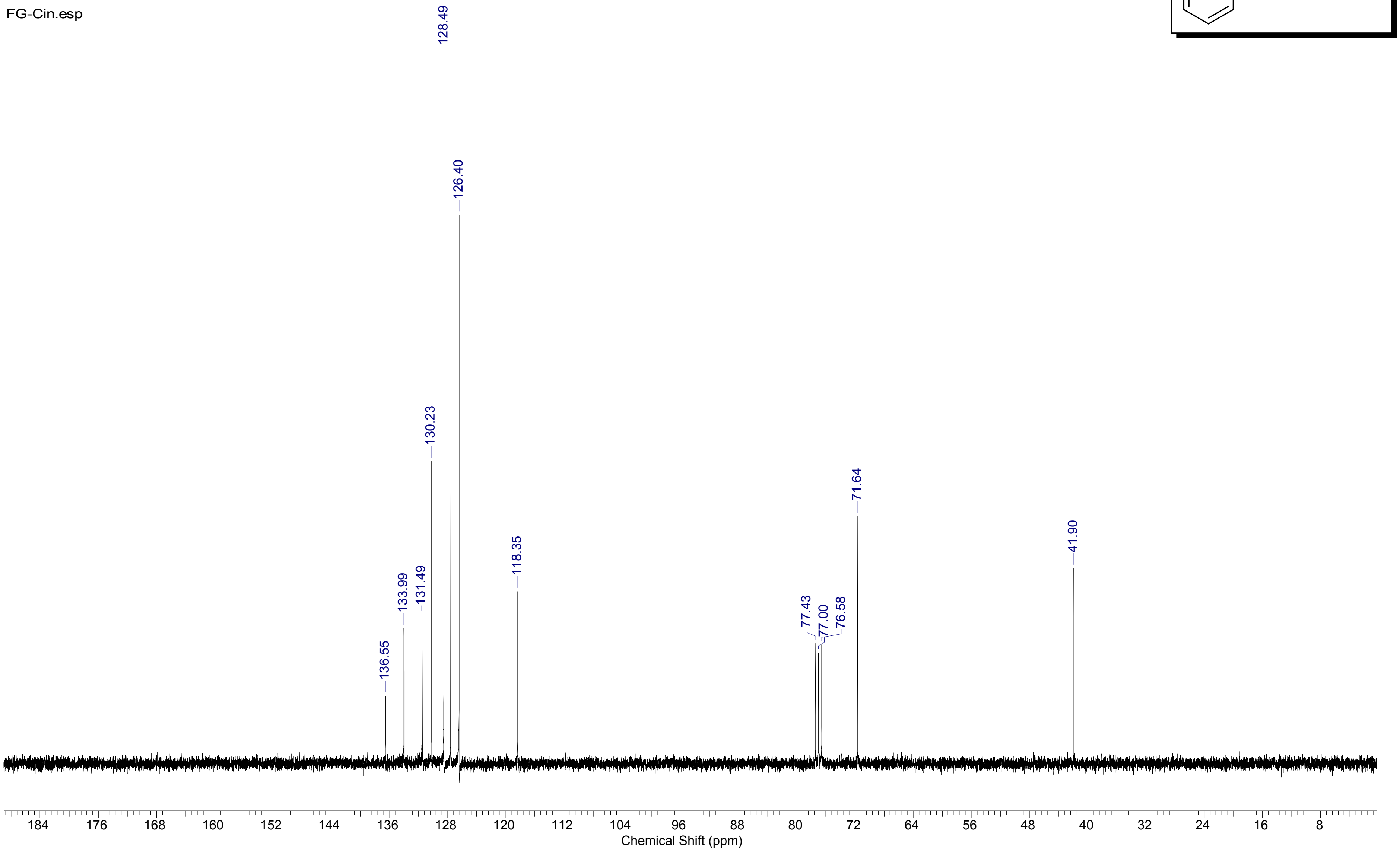

**Figure S17.** $^{1}$H-NMR spectrum (300 MHz, $CDCl_3$) of **3i**.

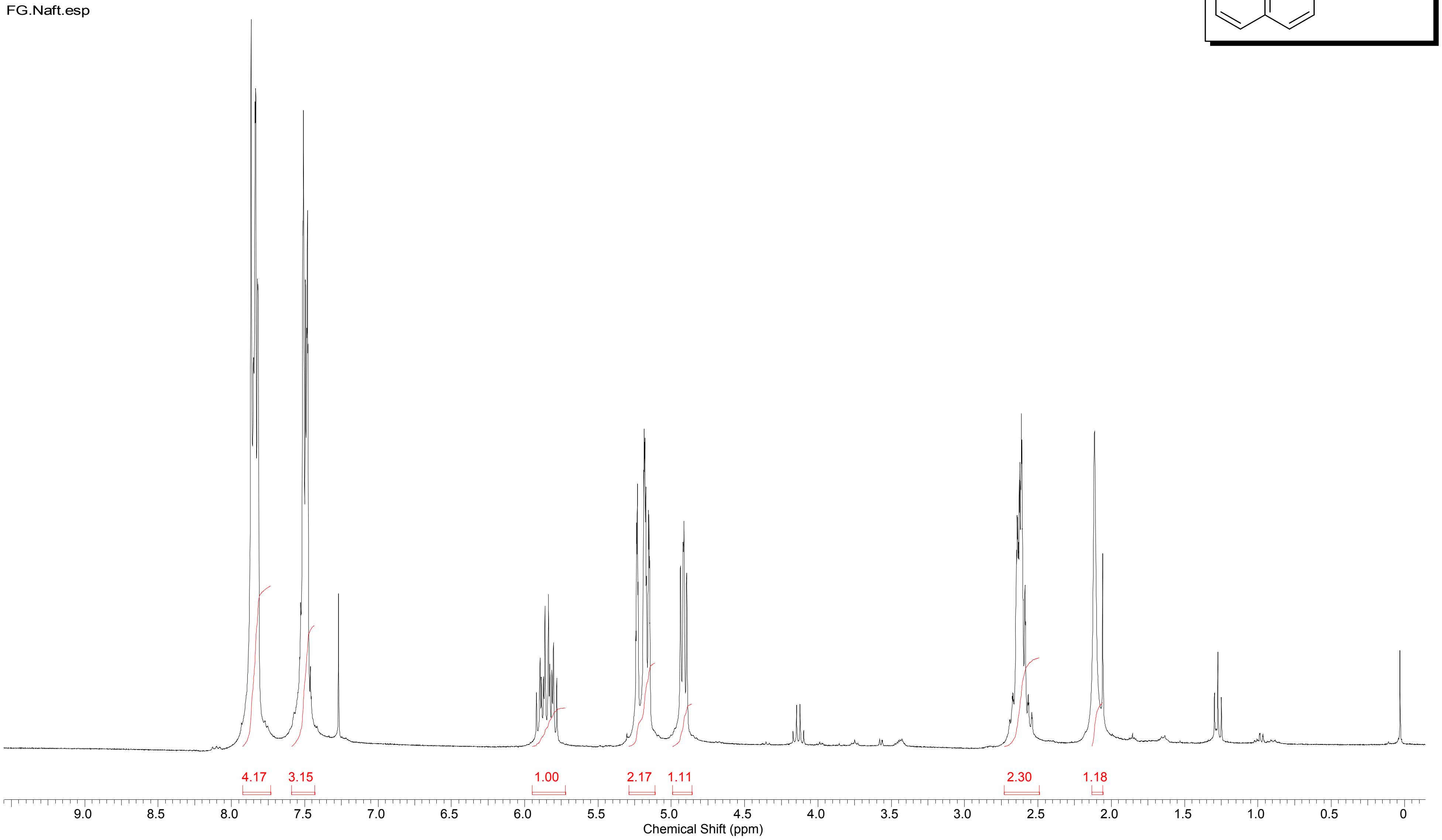


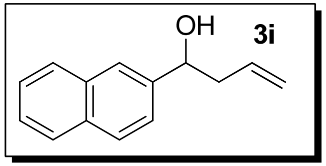

**Figure S18.** $^{13}$C-NMR spectrum (75 MHz, $CDCl_3$) of **3i**.

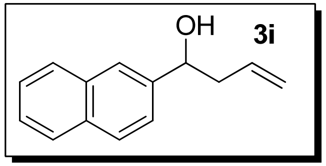


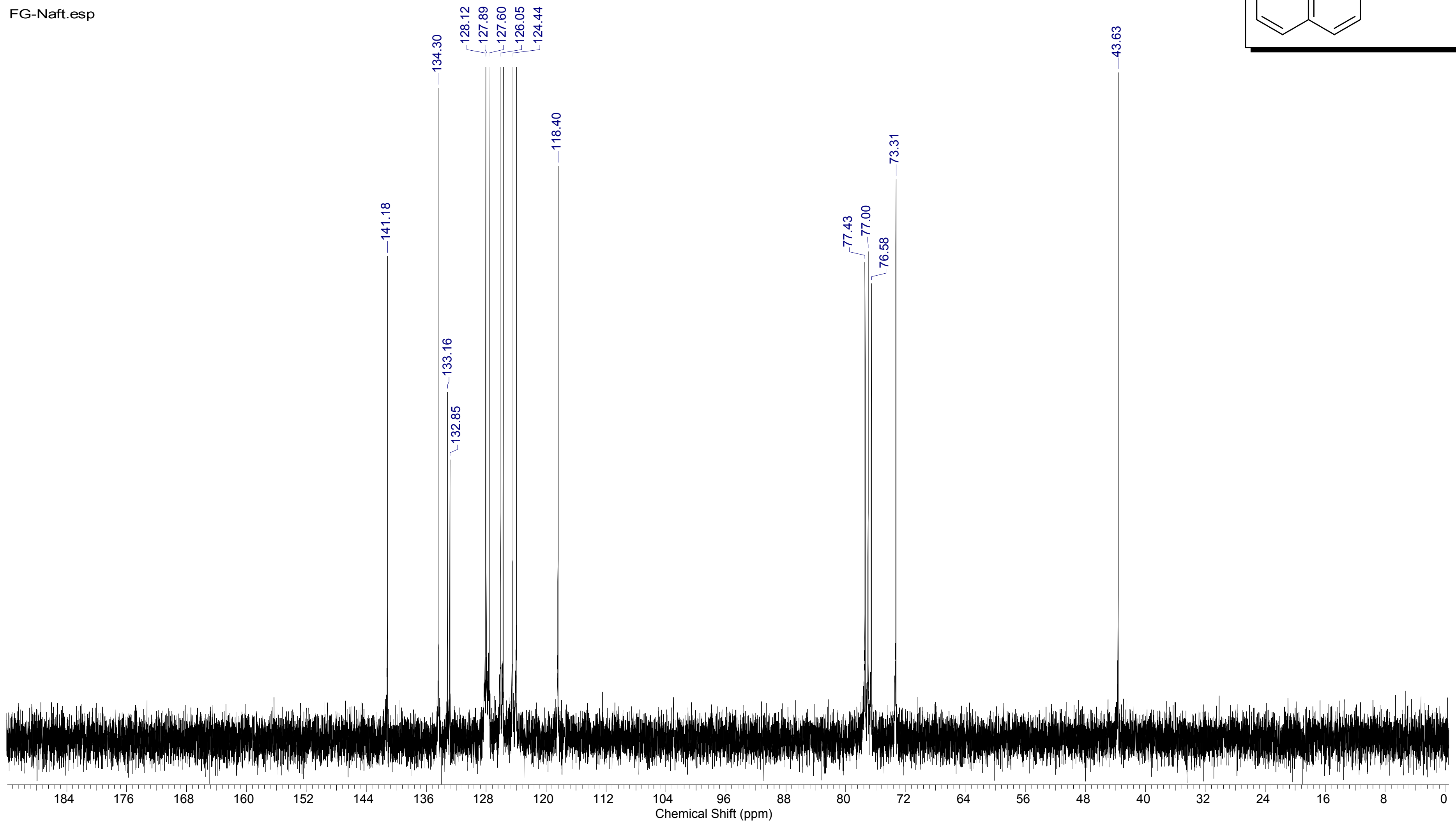

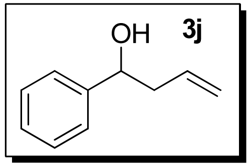


**Figure S19.** $^{1}$H-NMR spectrum (300 MHz, $CDCl_3$) of **3j.**

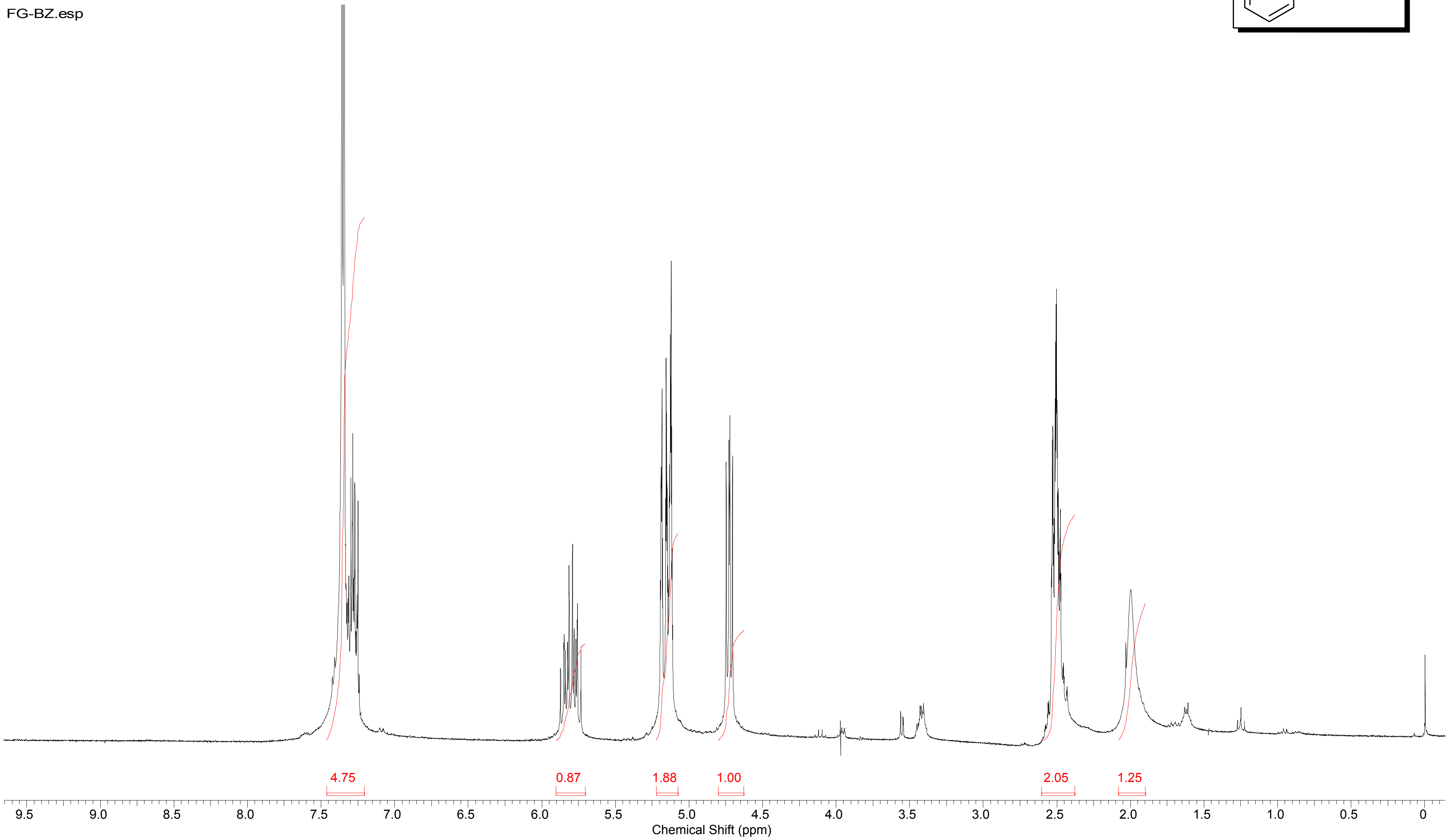

**Figure S20.** $^{13}$C-NMR spectrum (75 MHz, $CDCl_3$) of **3j**.

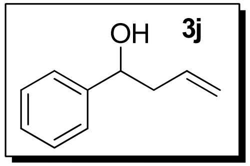


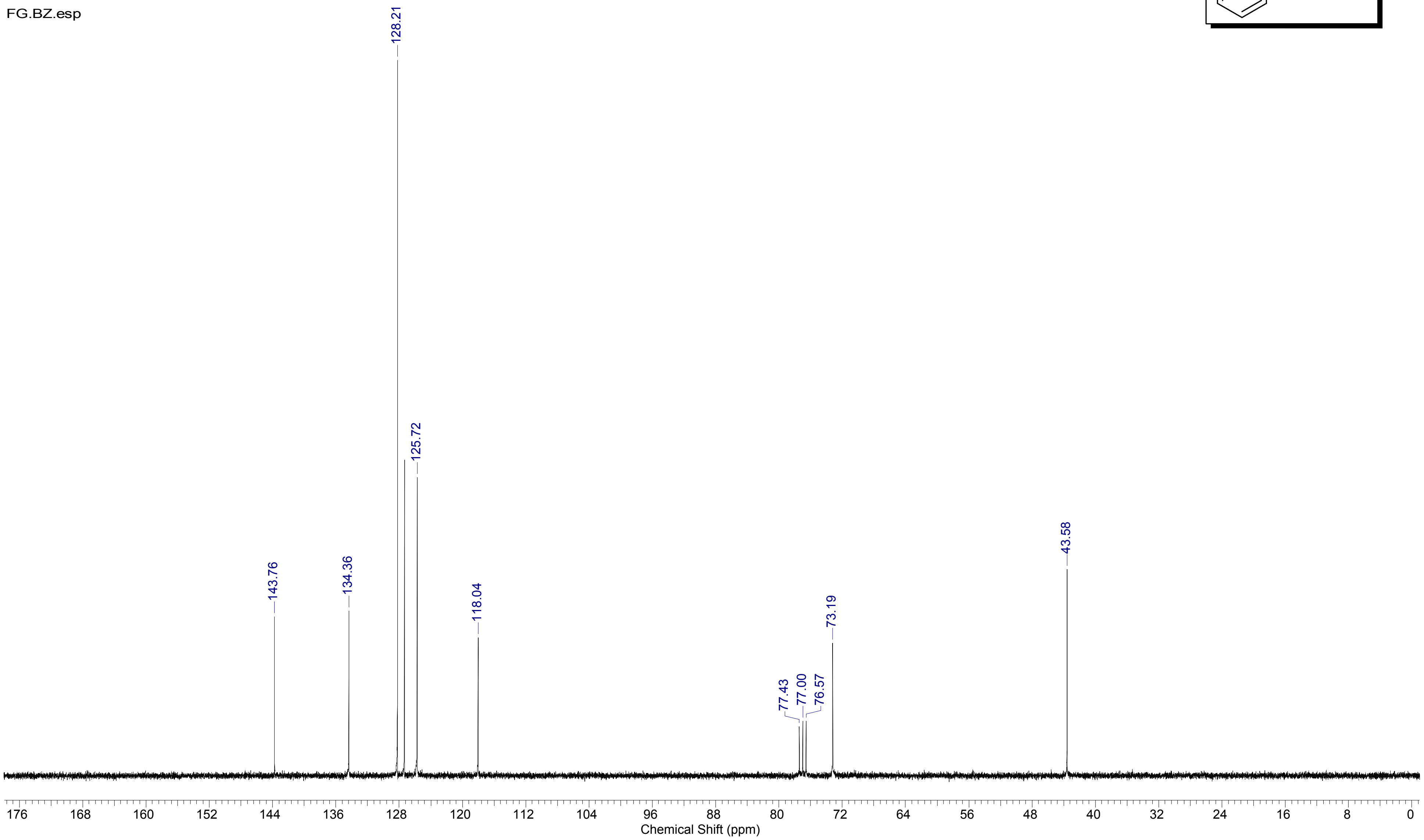

**Figure S21.** $^{1}$H-NMR spectrum (300 MHz, $CDCl_3$) of **3k**.

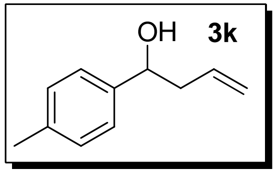


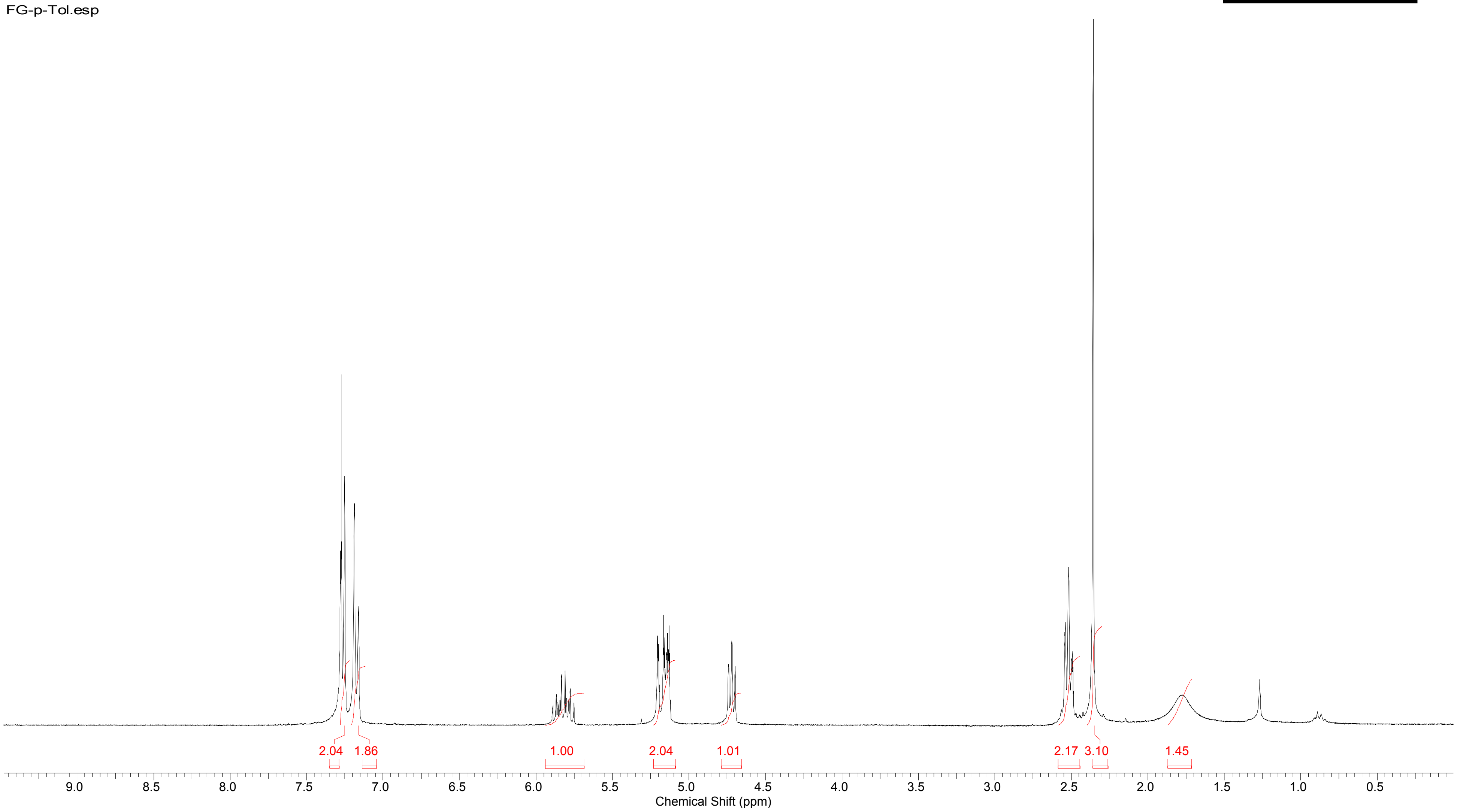

**Figure S22.** $^{13}$C-NMR spectrum (75 MHz, $CDCl_3$) of **3k**.

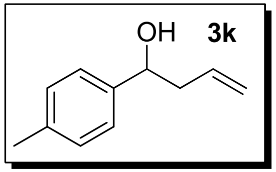


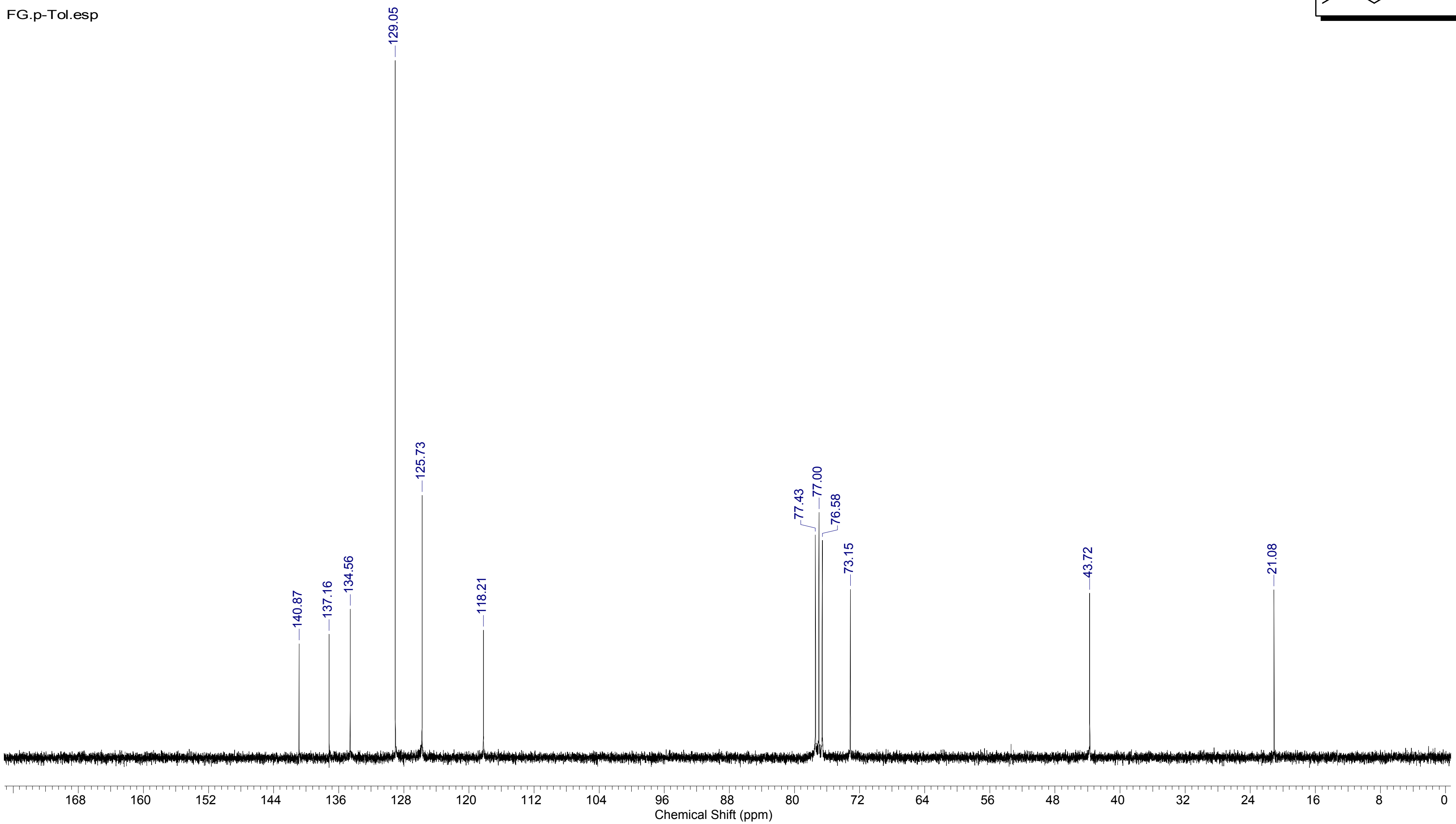

**Figure S23.** $^1$H-NMR spectrum (300 MHz, $CDCl_3$) of **3l**.

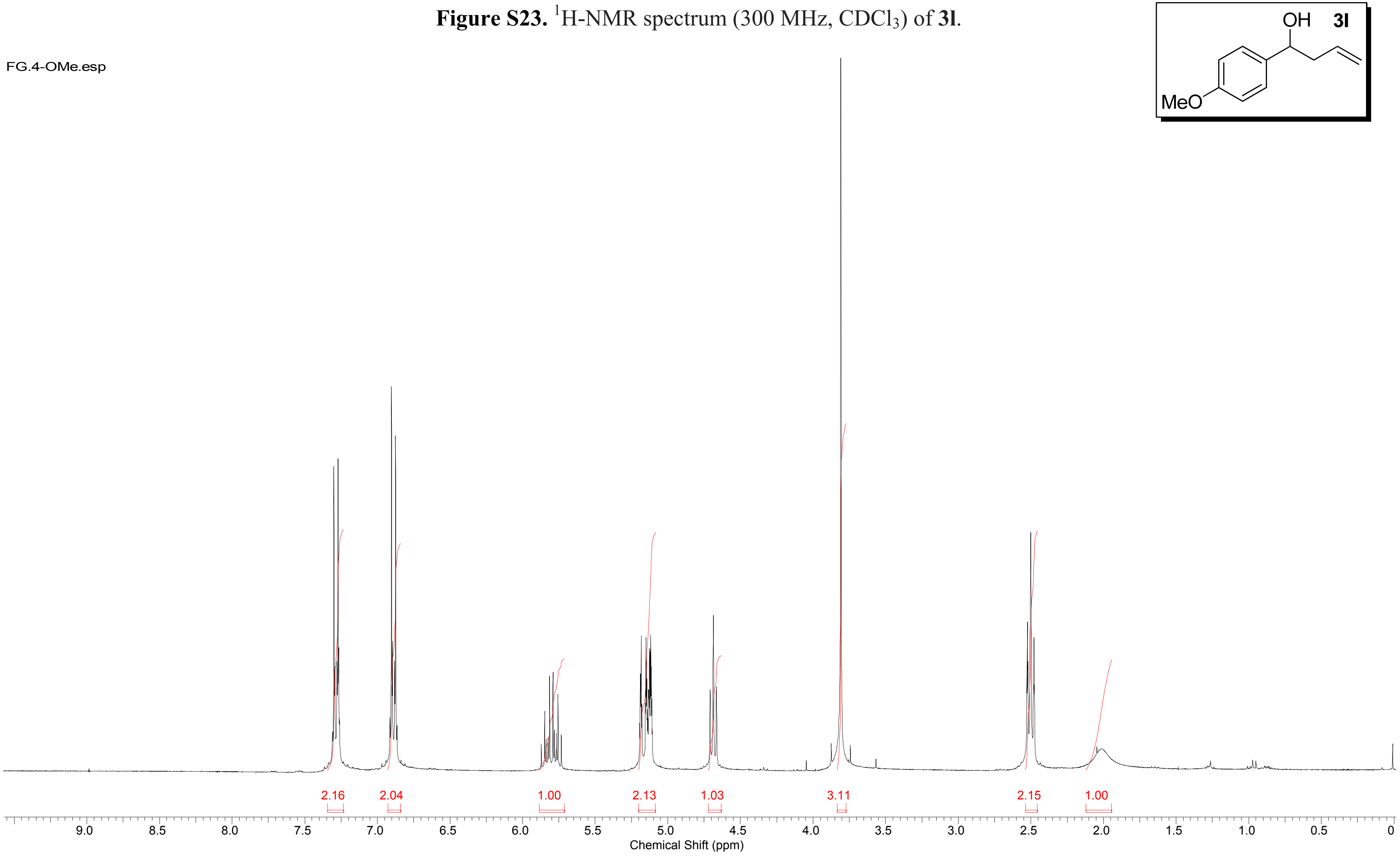

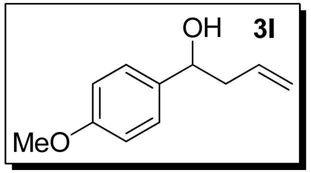

**3l**

**Figure S24.** $^{13}$C-NMR spectrum (75 MHz, $CDCl_3$) of **3l**.

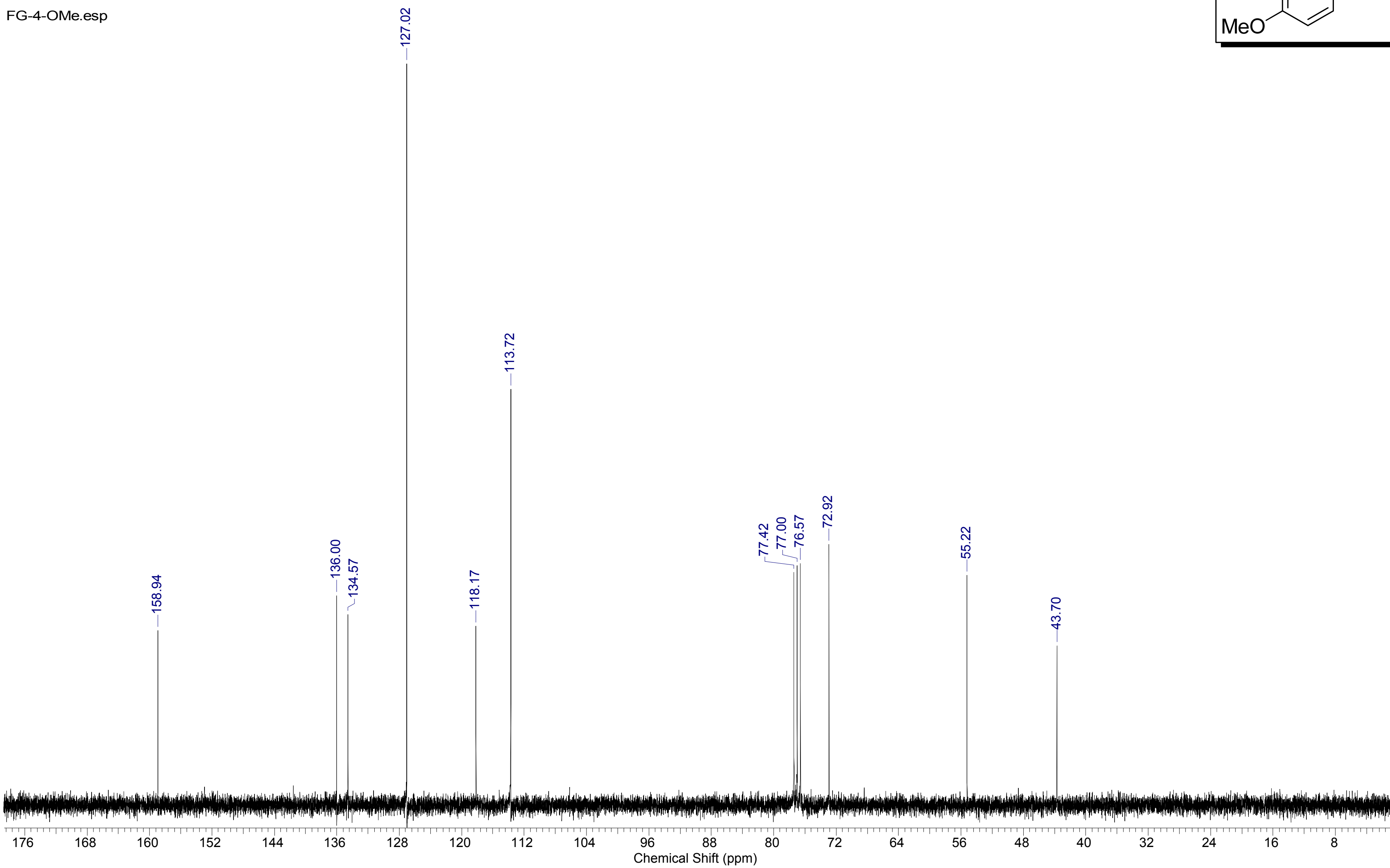

**Figure S25.** $^{1}H$-NMR spectrum (300 MHz, $CDCl_3$) of **3m**.

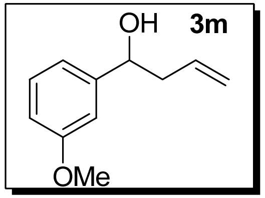


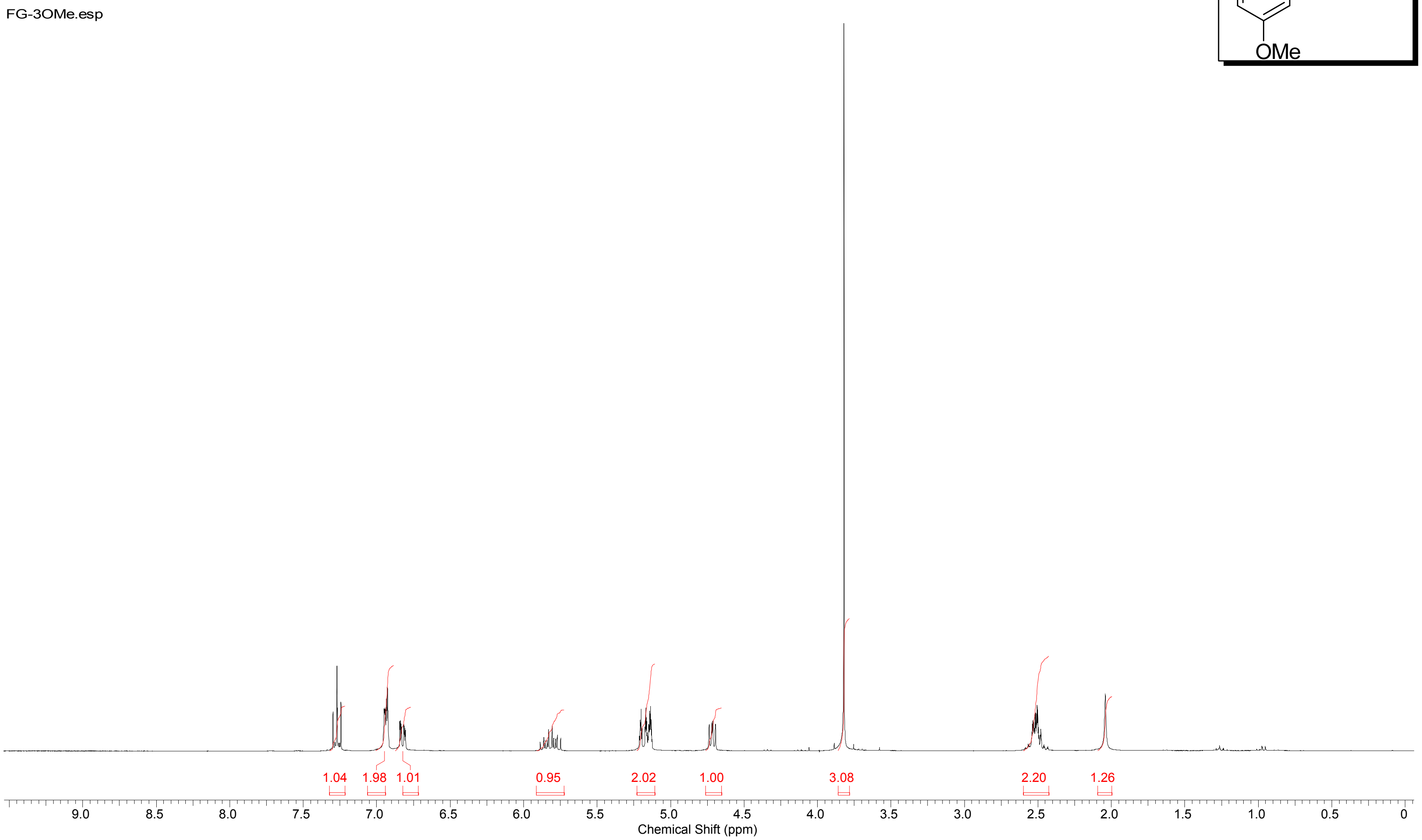

**Figure S26.** $^{13}$C-NMR spectrum (75 MHz, $CDCl_3$) of **3m**.

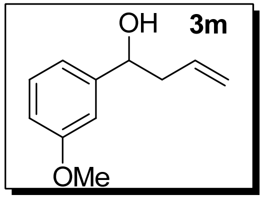


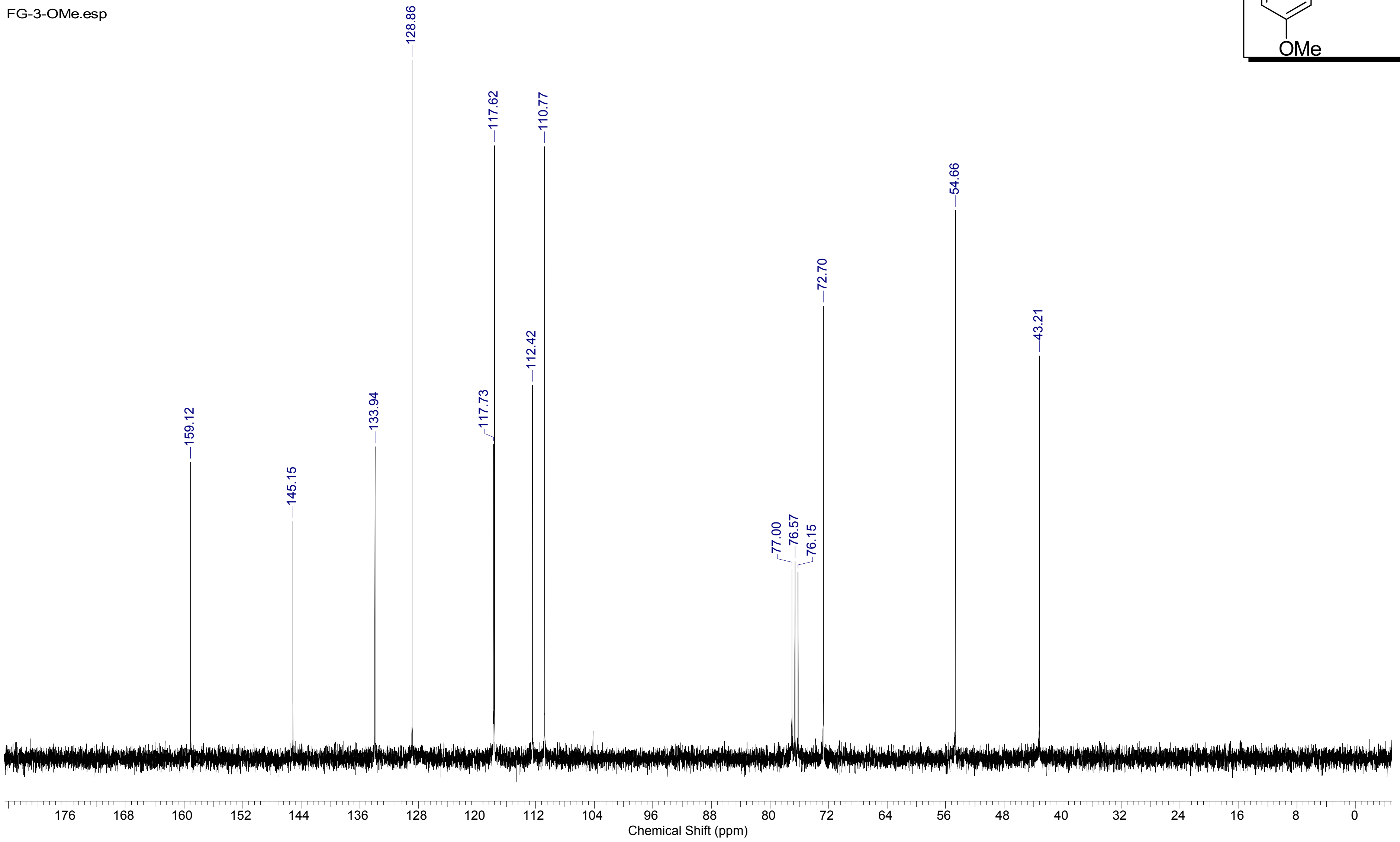

**Figure S27.** $^{1}$H-NMR spectrum (300 MHz, $CDCl_3$) of **3n**.

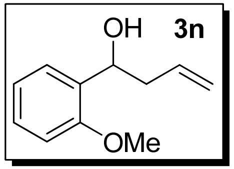


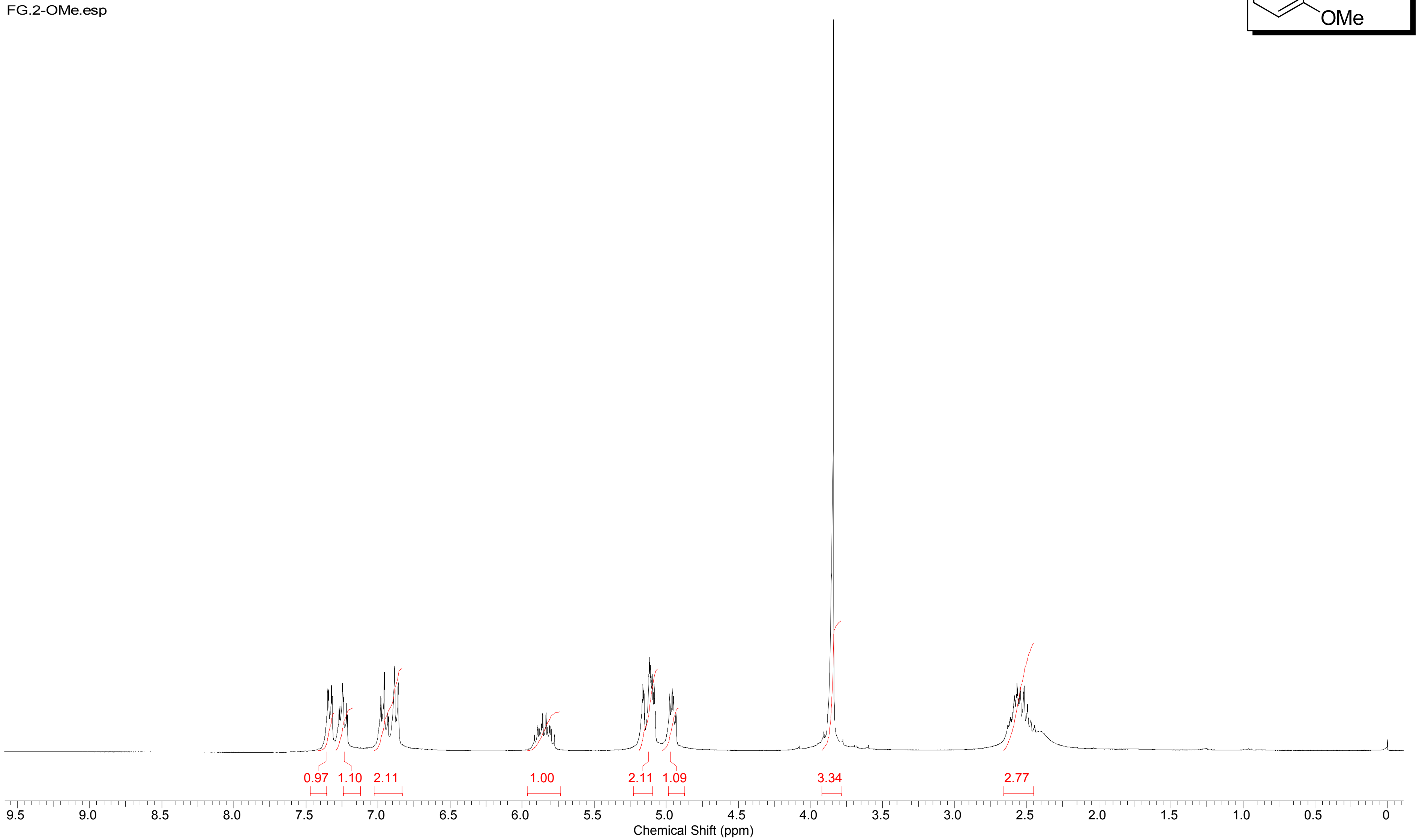

**Figure S28.** $^{13}$C-NMR spectrum (75 MHz, $CDCl_3$) of **3n**.

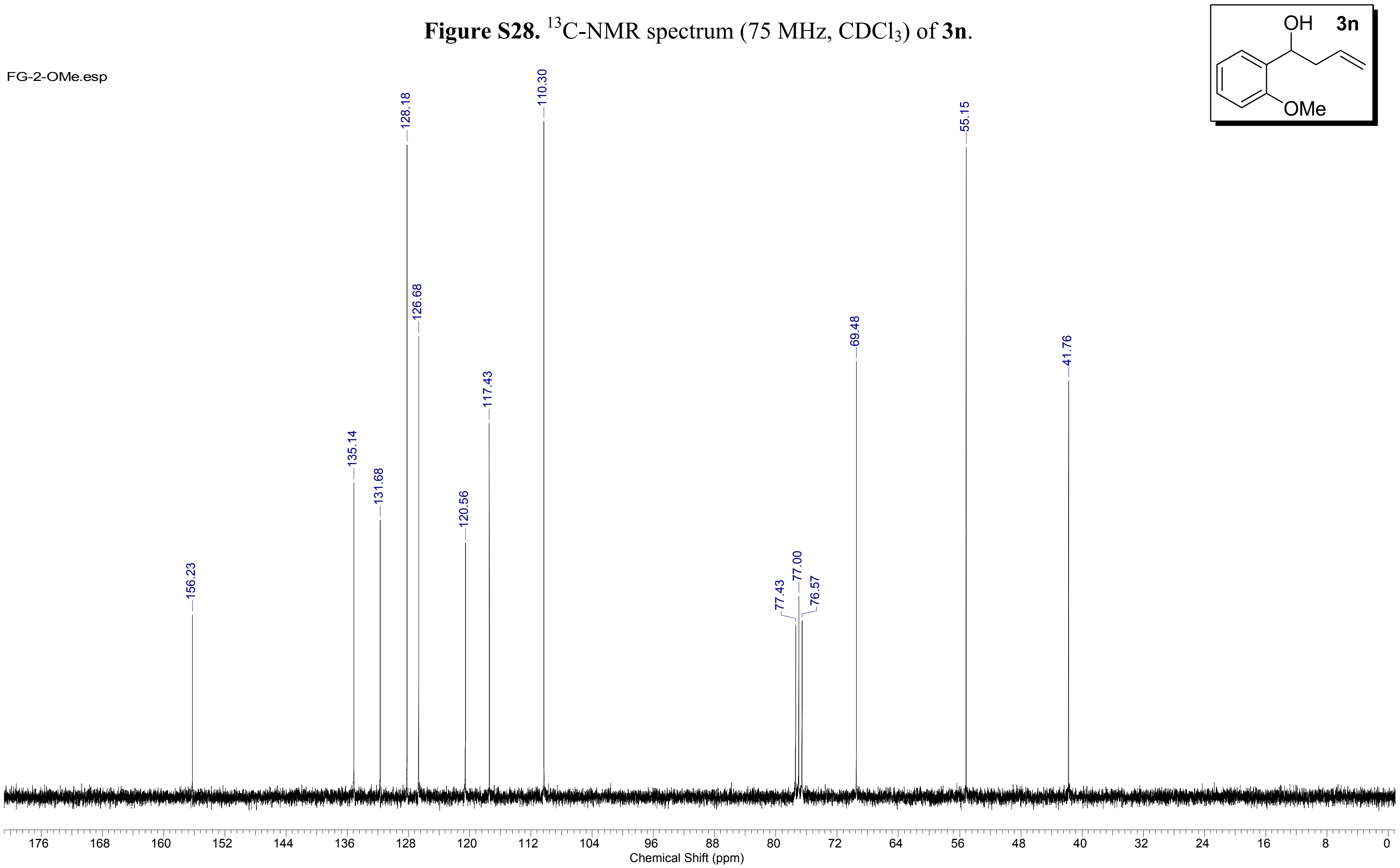

**Figure S29.** $^{1}$H-NMR spectrum (300 MHz, $CDCl_3$) of **3o**.

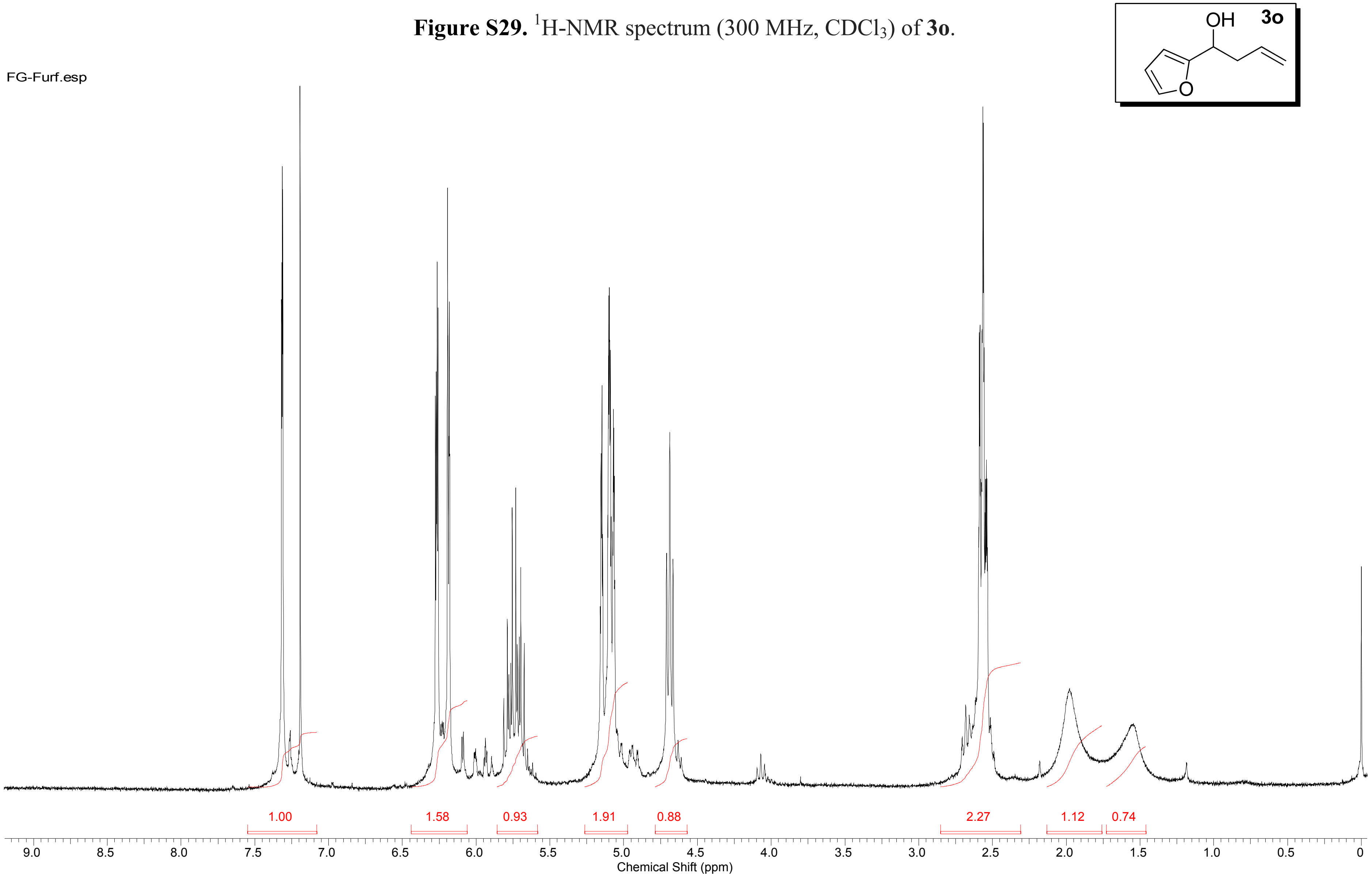

**Figure S30.** $^{13}$C-NMR spectrum (75 MHz, $CDCl_3$) of **3o**.

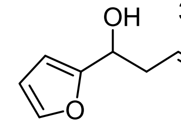

3o

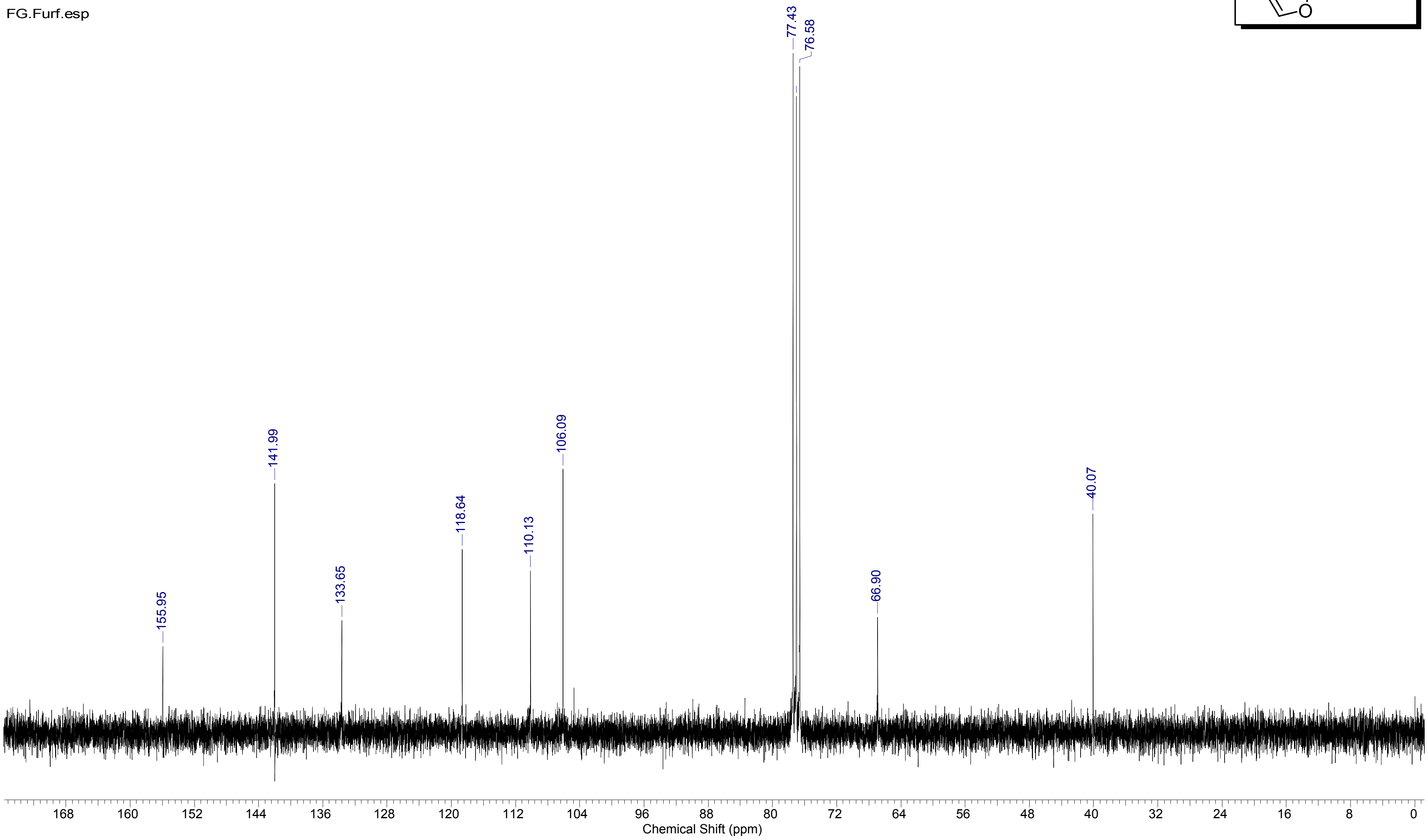

**Figure S31.** $^{1}$H-NMR spectrum (300 MHz, $CDCl_3$) of **5**.

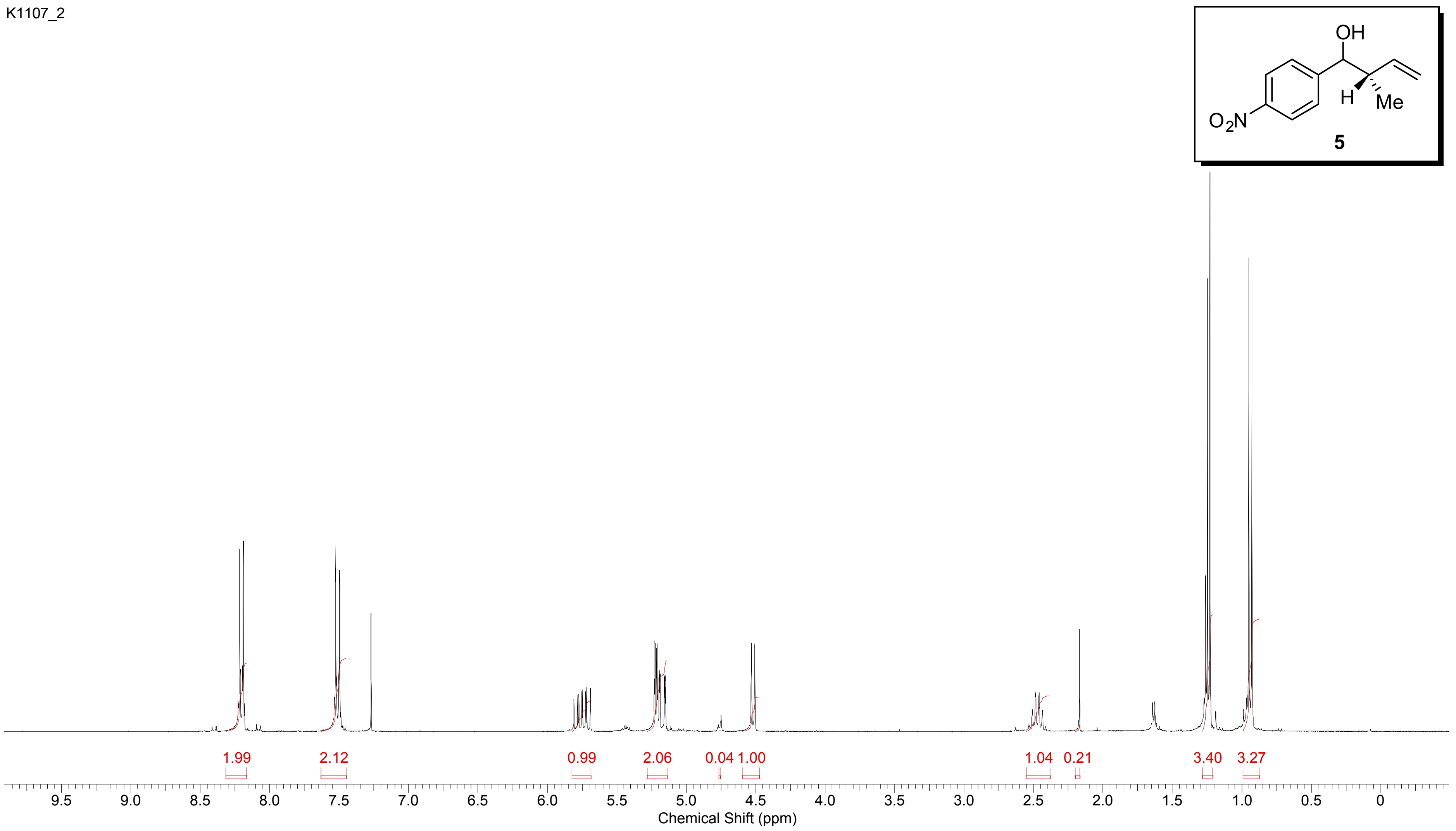

**Figure S32.** $^{1}$H-NMR spectrum (300 MHz, $CDCl_3$) of **6**.

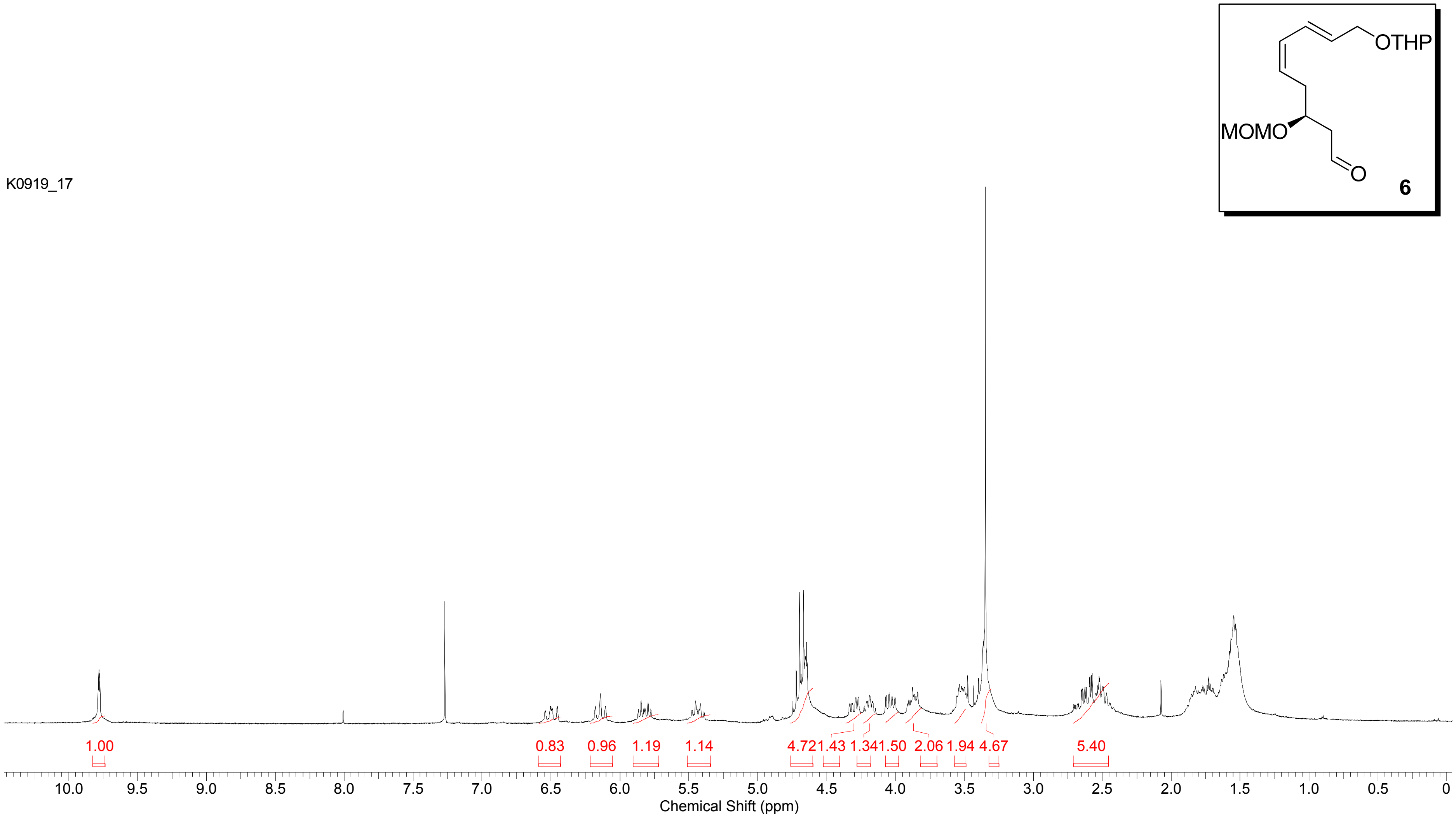

**Figure S33.** $^{13}$C-NMR spectrum (75 MHz, $CDCl_3$) of **6**.

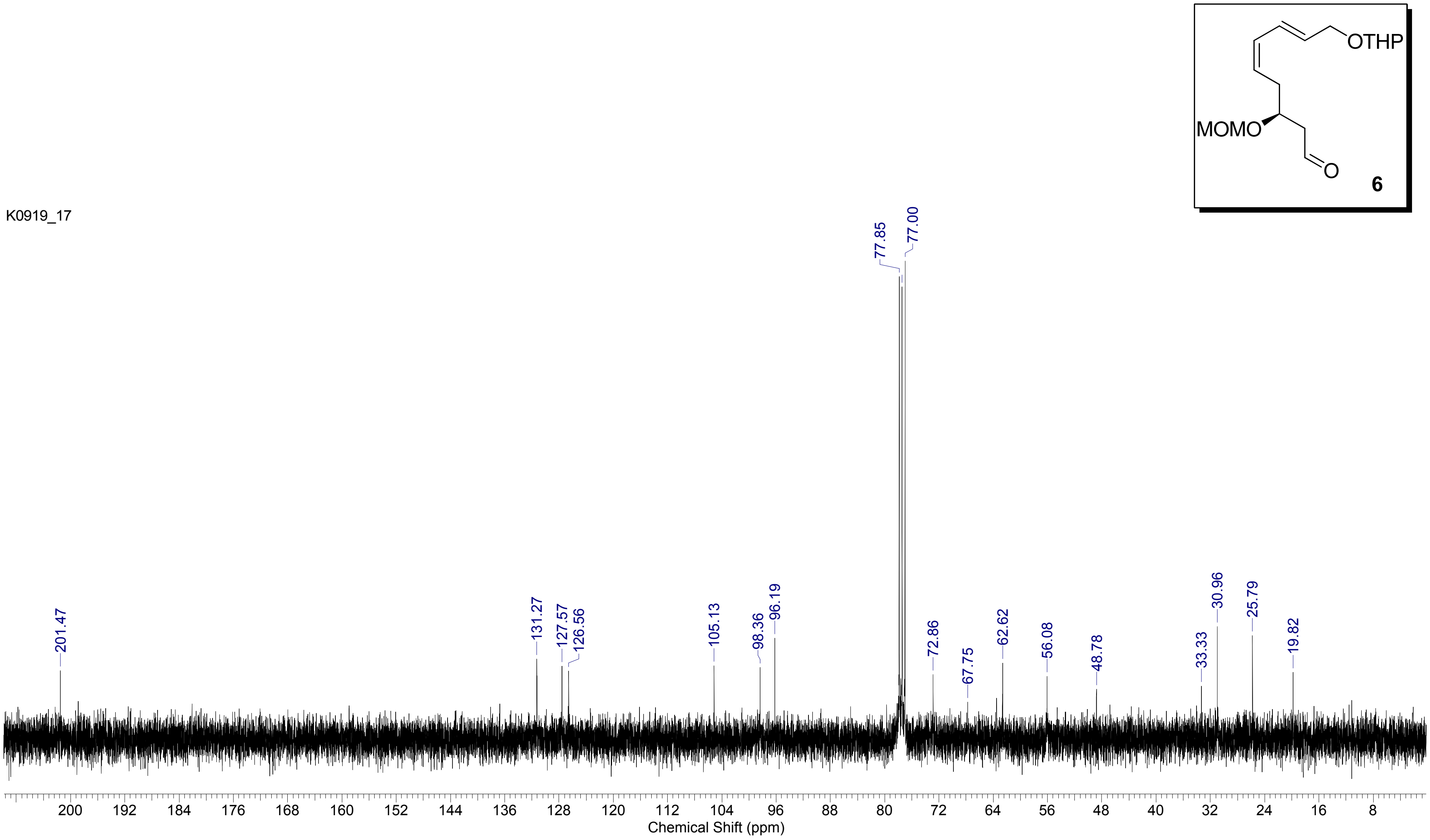

**Figure S34.** $^{1}$H-NMR spectrum (300 MHz, $CDCl_3$) of **7.**

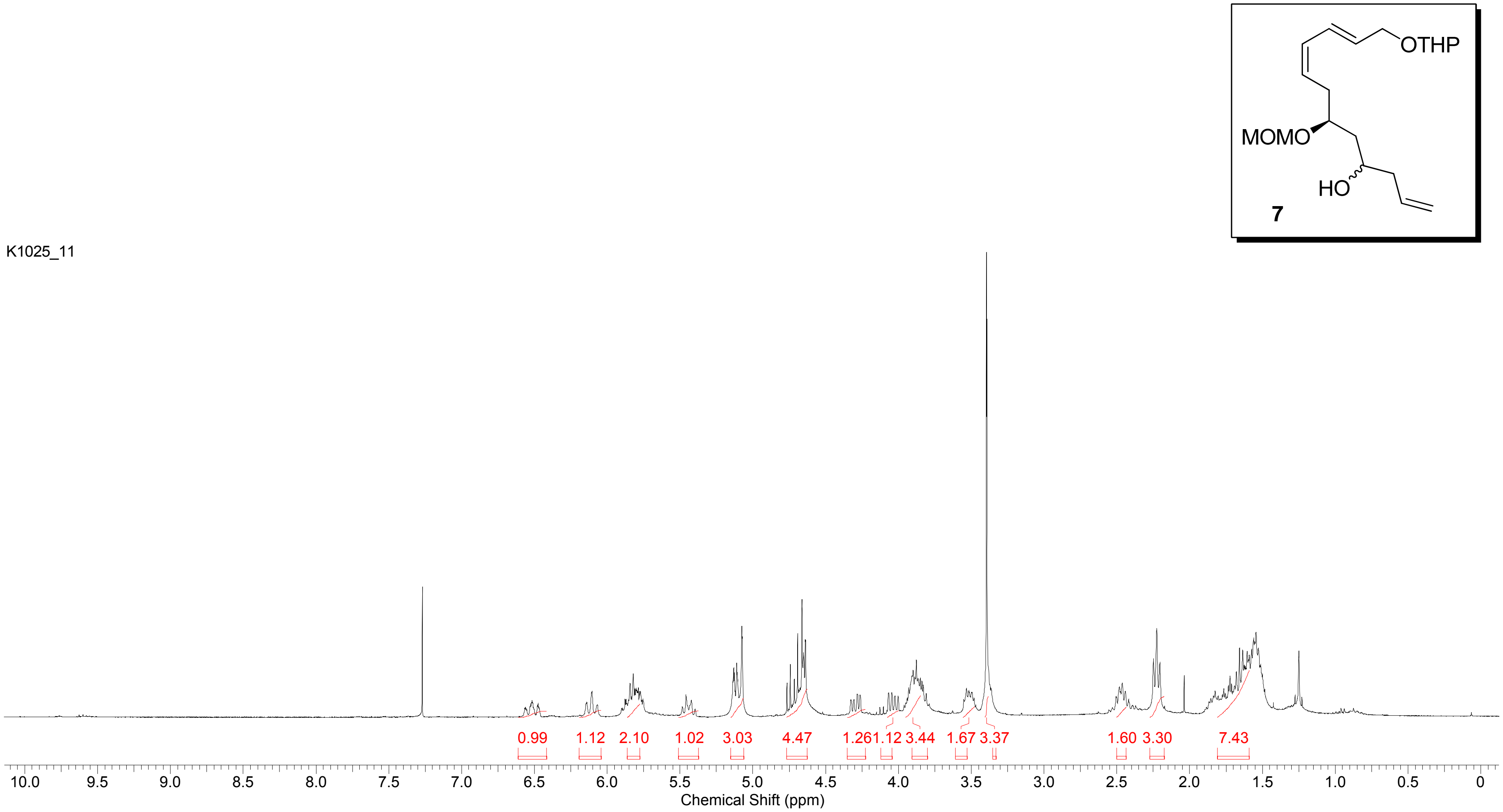

**Figure S35.** $^{13}$C-NMR spectrum (75 MHz, $CDCl_3$) of **7**.

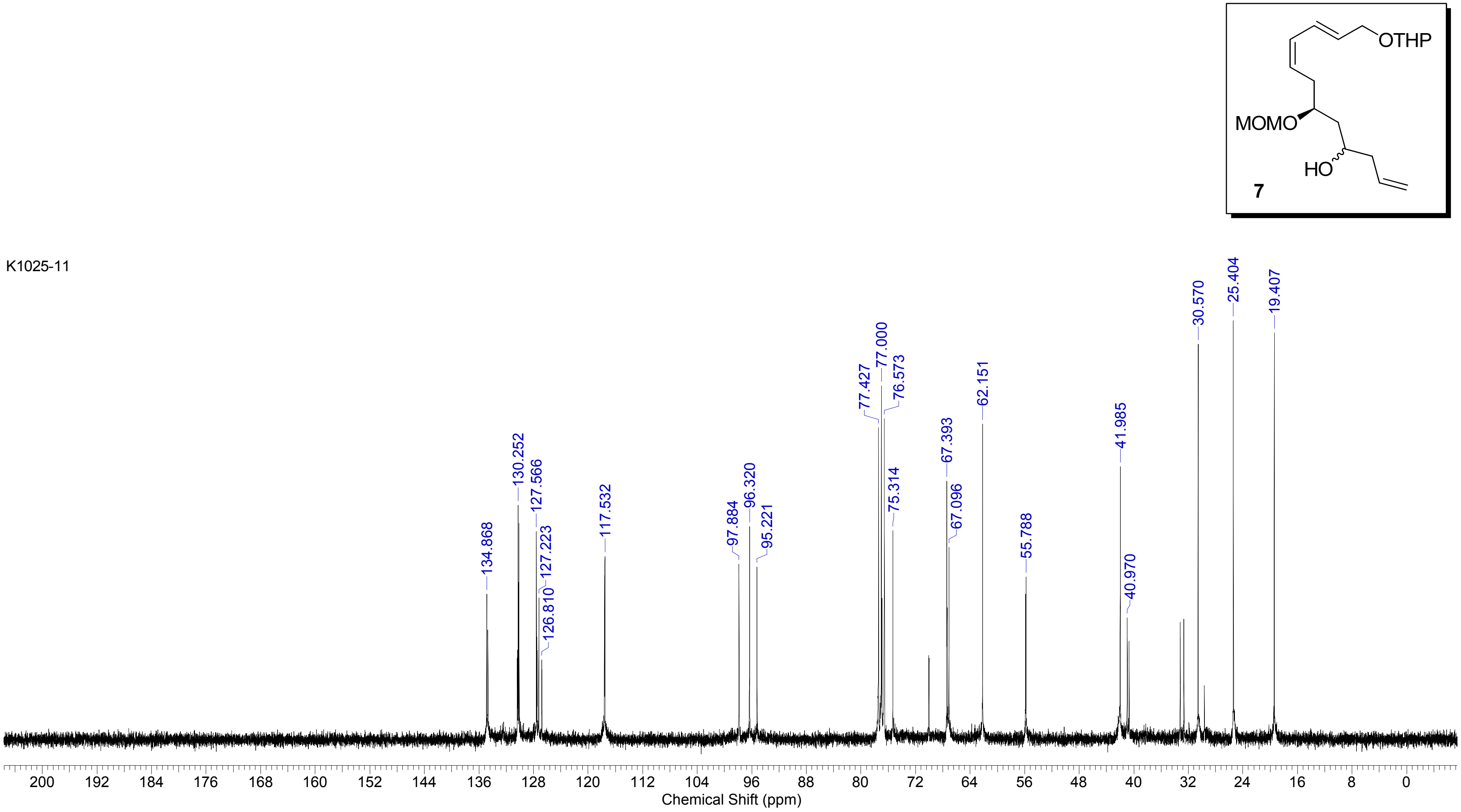

Supplement: Supplementary file 1 [file molecules-17-14099-s001.pdf]
